# GC-MS Based Metabolomics and NMR Spectroscopy Investigation of Food Intake Biomarkers for Milk and Cheese in Serum of Healthy Humans

**DOI:** 10.3390/metabo8020026

**Published:** 2018-03-23

**Authors:** Alessia Trimigno, Linda Münger, Gianfranco Picone, Carola Freiburghaus, Grégory Pimentel, Nathalie Vionnet, François Pralong, Francesco Capozzi, René Badertscher, Guy Vergères

**Affiliations:** 1 Department of Agricultural and Food Sciences (DISTAL), University of Bologna, 47521 Cesena, Italy; alessia@food.ku.dk (A.T.); gianfranco.picone@unibo.it (G.P.); francesco.capozzi@unibo.it (F.C.); 2 Agroscope, 3003 Berne, Switzerland; muenger.linda@gmail.com (L.M.); carola.freiburghaus@agroscope.admin.ch (C.F.); gregory.pimentel@agroscope.admin.ch (G.P.); rene.badertscher@agroscope.admin.ch (R.B.); 3 Service of Endocrinology, Diabetes and Metabolism, Lausanne University Hospital, 1011 Lausanne 1005, Switzerland; Nathalie.Vionnet@chuv.ch (N.V.); francois.pralong@latour.ch (F.P.)

**Keywords:** biomarker, metabolomics, nutrition, serum metabolome, milk, cheese, soy drink

## Abstract

The identification and validation of food intake biomarkers (FIBs) in human biofluids is a key objective for the evaluation of dietary intake. We report here the analysis of the GC-MS and 1H-NMR metabolomes of serum samples from a randomized cross-over study in 11 healthy volunteers having consumed isocaloric amounts of milk, cheese, and a soy drink as non-dairy alternative. Serum was collected at baseline, postprandially up to 6 h, and 24 h after consumption. A multivariate analysis of the untargeted serum metabolomes, combined with a targeted analysis of candidate FIBs previously reported in urine samples from the same study, identified galactitol, galactonate, and galactono-1,5-lactone (milk), 3-phenyllactic acid (cheese), and pinitol (soy drink) as candidate FIBs for these products. Serum metabolites not previously identified in the urine samples, e.g., 3-hydroxyisobutyrate after cheese intake, were detected. Finally, an analysis of the postprandial behavior of candidate FIBs, in particular the dairy fatty acids pentadecanoic acid and heptadecanoic acid, revealed specific kinetic patterns of relevance to their detection in future validation studies. Taken together, promising candidate FIBs for dairy intake appear to be lactose and metabolites thereof, for lactose-containing products, and microbial metabolites derived from amino acids, for fermented dairy products such as cheese.

## 1. Introduction

Food intake biomarkers (FIBs) are defined as specific metabolites that reflect the consumption of one specific food or food group, show a clear time- and dose-response and are ideally not present if the food has not been ingested [1]. Their measurement in biofluids such as serum, plasma, and urine is intended to be used as a complement to current dietary assessment methods as, for instance, food frequency questionnaires, 24 h-recalls, and food records [2] all of them being highly prone to bias due to self-reporting [3,4]. By being indicative of the intake of specific foods, ideally among a broad range of other foods, FIBs can be applied to assess dietary intake in an objective way. In this context, dietary intervention studies allow identifying candidate FIBs for selected food items under controlled conditions.

Blood serum is a standard biological system employed to investigate postprandial changes in metabolite composition in a time-related manner. Going beyond the analysis of classical blood parameters such as glucose and total triglycerides, untargeted metabolomics approaches using liquid chromatography-mass spectrometry (LC-MS), gas chromatography-mass spectrometry (GC-MS), or nuclear magnetic resonance (NMR) allow to measure a large number of low-molecular-weight metabolites of different chemical classes (amino acids, sugars, lipids, organic acids, etc.) within one single analysis [5]. These untargeted metabolomics methods in combination with multivariate data analysis can thus be applied to identify FIBs [6,7]. The candidate FIBs found by dietary intervention studies, then need to be validated in a second step under free-living conditions before being finally used in epidemiological studies [7,8,9].

The Joint Programming Initiative ‘A Healthy Diet for a Healthy Life’ (JPI-HDHL) Food Biomarkers Alliance (FoodBAll) aims to promote FIB research [10,11,12]. To contribute to this research area, seven study centres are presently conducting well-defined standardised short-term intervention studies. All study centres follow the same design, exploring potential food intake biomarkers of fourteen foods, including sugar-sweetened beverage, apple, tomato, banana, bread, meat and meat products, potato, carrot, peas, lentils, beans, chickpeas, milk, and cheese.

In light of their relevance in the diet of many human populations, the identification of validated FIBs for dairy products is an important goal. In that context, serum short chain fatty acids were increased after daily milk intake over a period of seven days [13]. Data from several observational studies [14,15,16,17] and few intervention studies [18,19] obtained by targeted analyses has suggested that serum pentadecanoic acid (C15:0) and heptadecanoic acid (C17:0), mostly in form of cholesteryl esters or phospholipids, are potential markers for dairy fat intake. Odd chain fatty acids occur at low amounts in ruminant fat but also in other foods [20]. A recent study showed that only C15:0 directly correlates with ruminant fat intake while increasing evidence suggests that C17:0 can be biosynthesized in vivo [21]. Non-lipid molecules have also been proposed as markers for the intake of dairy products and Pedersen and Nebel [22] showed an increase of serum lactate and several amino acids after 8 weeks of daily intake of acidified milk with or without probiotics when compared to baseline. Increased lactate level may be attributed to the acidification of the milk while amino acids are not specific for milk intake. Furthermore, urine samples have been analysed for the identification of candidate FIBs for dairy products such as creatinine for milk and 4-hydroxyphenylacetic acid for cheese [23,24]. Taken together, however, FIBs for dairy products have not been established yet and additional well-designed studies are requested to identify and, eventually, validate such FIBs. This report presents the effort of FoodBAll in identifying candidate serum FIBs for dairy products, in particular milk and cheese.

We recently demonstrated in a randomized, cross-over study with soy drink as a non-dairy alternative, that compounds deriving from lactose/galactose metabolism, in particular, are reflective of milk intake and that 3-phenyllactic acid was a specific candidate urinary FIB for cheese [25]. Serum samples, at time-point 0 h and postprandially up to 24 h (at 1, 2, 4, 6, and 24 h), were also collected in this acute intervention study, in addition to urine samples. The aim of the present study was to identify candidate FIBs of milk and cheese in serum using untargeted GC-MS and NMR to gain insight in their kinetics, and to compare them to the urinary FIBs that have been identified previously. This untargeted approach was complemented by a targeted search of the metabolites already identified as candidate FIBs in the urine samples of the same study [25]. In addition, the dairy specific fatty acids C15:0 and C17:0 were studied in serum in more detail using a targeted approach due to their potential role as dairy fat biomarker.

## 2. Results

### 2.1. GC-MS

After deconvolution, 2014 features appeared in the chromatograms after 10 min elution. Features eluting prior to 10 min were not included as excess reagents eluted in this region. Filtering this dataset by removing all features, which did not demonstrate a statistically significant postprandial response after the intake of at least one food group (nparLD test, *p* < 0.05 for time effect), decreased it to 526 features. A Projections to Latent Structures Discriminant Analysis (PLS-DA) model was built with this filtered dataset and using the 6h incremental areas under the curve (6h-iAUC). The PLS-DA analysis of the 33 iAUCs (3 food groups for each of the 11 subjects) showed a separation of the food groups (R2 = 0.89; Q2 = 0.61), the milk and cheese groups showing some overlap (Figure S1). Interestingly, a PLS—DA model defining time-points as classes revealed the postprandial dynamics and showed that the 0 and 24 h samples, both groups collected in the fasted state, were overlapping (Figure S2) suggesting that, overall, the 24 h-samples did not add value to the marker detection.

Orthogonal Projections to Latent Structures Discriminant Analysis (OPLS-DA) models were then built with the 6h-iAUCs (1 by 2 comparison) for biomarkers selection. A summary of the three OPLS-DA models is shown in Figures S3–S5 for milk, cheese, and the soy drink respectively. These models were characterized with R2 > 0.95 and Q2 > 0.53, thus indicating good predictability. The highest Q2 was obtained when comparing soy drink intake against cheese and milk intake (0.77), followed by the cheese intake model (0.62), and the milk intake model (0.53). Marker compounds were further selected based on a Variable Importance for the Projection (VIP) threshold value of 2.0 (Table S1). A targeted analysis of additional candidate FIBs was also conducted, whose selection was based on information available in the literature, in particular on the results of the urine analysis of the same study [25] (Table S2). The 6h-iAUC was used for all markers excepted for guaiacol and catechol for which the 24h-iAUC was used in light of the late appearance of these metabolites in the urine samples of this study [25]. A Kruskal-Wallis test of the individual time-points for each of the selected candidate markers revealed that most of them can be detected postprandially at more than one time-point between 1 and 6 h (lactose, galactose, galactitol, galactonate, galactono-1,5-lactone, methionine, proline, 3-phenyllactic acid, dodecanoic acid, linoleic acid, γ-tocopherol, maltol, sucrose, unknown S13, unknown S27), their presence in serum being no longer detected at 24 h (Table S3). The three exceptions among all these metabolites are the soy candidate markers pinitol, guaiacol, and catechol which can be detected at 24 h. Accordingly, a Conover-Inman test for multiple comparison for the candidate markers at each of the postprandial time-points 1, 2, 4, and 6 h indicated that most of these molecules can differentiate the three treatments in one by one comparisons (Table S4), the 24 h time-point being only discriminative for the soy candidate markers pinitol. However, In contrast to their measure in late postprandial urine pools [25], serum guaiacol and catechol could not significantly differentiate soy drink intake from dairy intake at 24 h. The candidate FIBs assessed by GC-MS for each of the three foods are presented in detail below.

#### 2.1.1. Candidate Fibs for Milk

**Figure 1:**
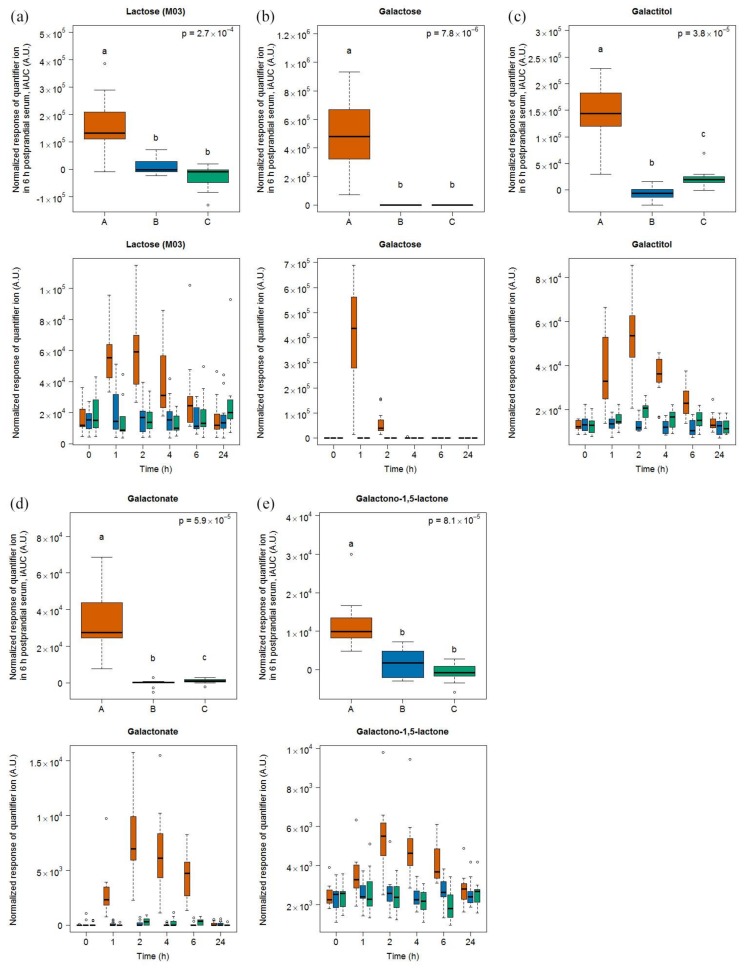
GC-MS-based postprandial behaviour of lactose (**a**), galactose (**b**), galactitol (**c**), galactono-1,5 lactone (**d**), and galactonate (**e**) after milk intake (orange) when compared to cheese (blue) and soy drink (green) intake. Upper graph of panels: 6h-iAUC. Lower graph of panels: time-points analysis from 0 to 24 h. The *p* values in the upper graphs are based on a Kruskal-Wallis test and have been adjusted for multiple testing. If the Kruskal-Wallis test was significant (*p* < 0.05), pairwise comparisons were conducted using a Conover-Inman test. Different letters (abc) denote significant differences based on a *p* value < 0.05.

After milk intake, lactose (eluting at three different retention times (RT) due to stereoisomer formation during derivatization), galactose, and galactonate were most discriminative based on multivariate analysis (see Figure S3). The highest response (M03: RI 2671), out of the three lactose peaks, was further evaluated using a targeted approach with its quantifier ion. Univariate analysis of 6h-iAUCs for lactose, galactose, and galactonate resulted in significant differences between their postprandial response after the intake of milk, cheese, and soy drink (Figure 1). Galactose and galactonate were exclusively detected after the intake of milk while lactose was also identified after cheese and soy drink intake, although its level was significantly increased after milk intake. Galactitol and galactono-1,5-lactone were indicative of milk intake in urine from samples collected within this study [22]. Although these two molecules were not identified by multivariate analysis as markers in serum, they were discriminating for milk intake in serum when analyzed in a targeted manner based on known quantifier and qualifier ion as well as RI and subsequent univariate analysis of their 6h-iAUC.

Galactose reached its highest serum levels 1 h after milk intake while lactose, galactitol, galactonate, and galactono-1,5-lactone reached their maximum at 2 h. At 4 h, galactose was below the limit of detection whereas the other milk markers were still at detectable levels and significantly higher than after the intake of the two other foods. Significant differences between the intake of the three foods after 6 h were found in case of galactonate, galactitol, and galactono-1,5-lactone (see Tables S3 and S4).

#### 2.1.2. Candidate Fibs for Cheese

**Figure 2:**
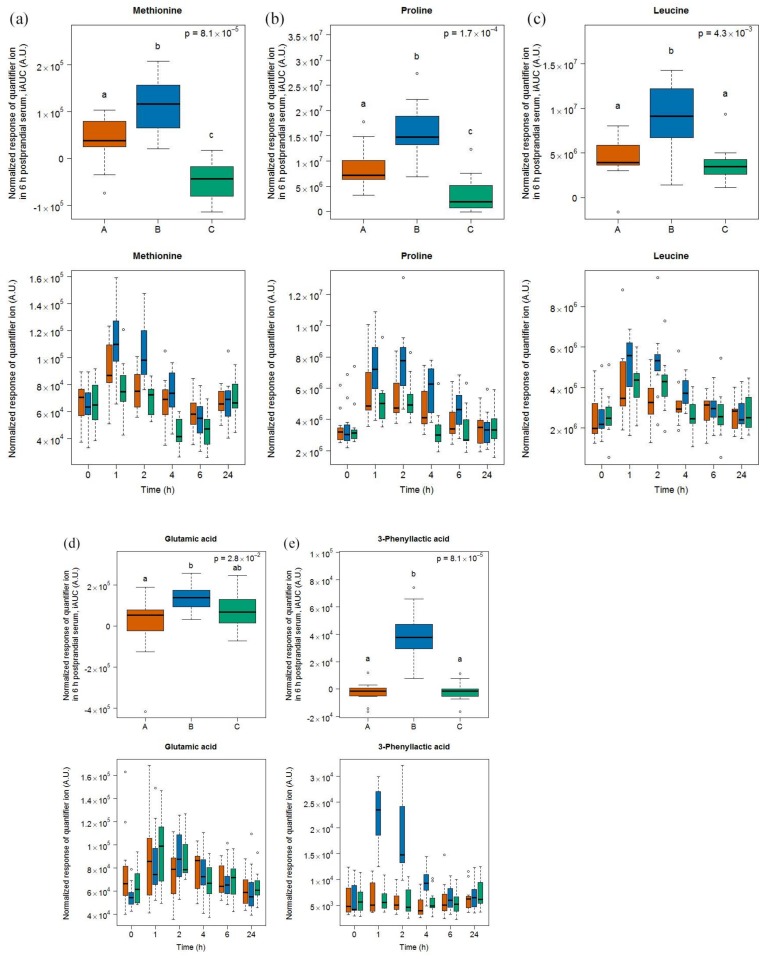
GC-MS-based postprandial behaviour of methionine (**a**), proline (**b**), leucine (**c**), glutamic acid (**d**), and 3-phenyllactic acid (**e**) after cheese intake (blue) when compared to milk (orange) and soy drink (green) intake. Upper graph of panels: 6h-iAUC; Lower graph of panels: time-points analysis from 0 to 24 h. The *p* values in the upper graphs are based on a Kruskal-Wallis test and have been adjusted for multiple testing. If the Kruskal-Wallis test was significant (*p* < 0.05), pairwise comparisons were conducted using a Conover-Inman test. Different letters (abc) denote significant differences based on a *p* value < 0.05.

After cheese intake, four amino acids were discriminating based on an OPLS-DA analysis of their 6h-iAUC (see Figure S4) and their subsequent targeted quantification, namely methionine, proline, leucine, and glutamic acid (Figure 2). Together with 3-phenyllatic acid, which was detected postprandially in urine after the intake of cheese [25], each of these metabolites was higher after cheese intake, compared to milk and soy drink.

3-phenyllactic acid showed significant differences to milk and soy drink intake at time-points 1, 2, and 4 h (see Tables S3 and S4). Likewise, methionine, proline, and leucine levels were significantly higher at these time-points after cheese intake although an increasing postprandial response was also observed after milk and soy drink intake.

#### 2.1.3. Candidate FIBs for Soy Drink

**Figure 3:**
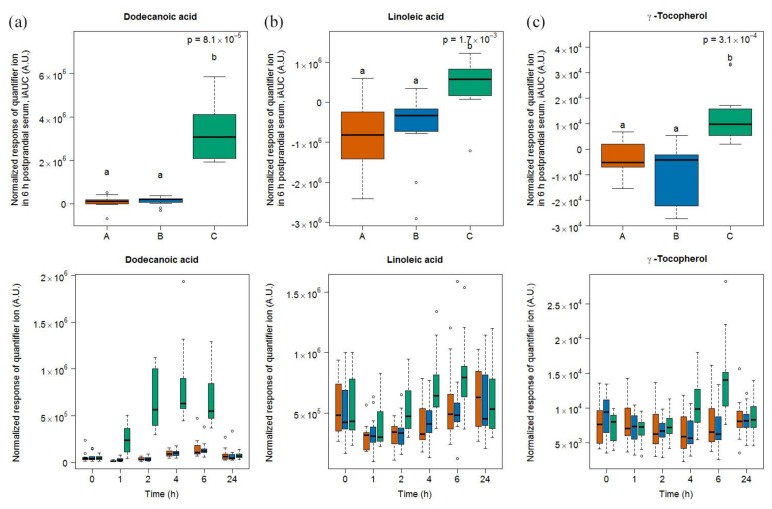

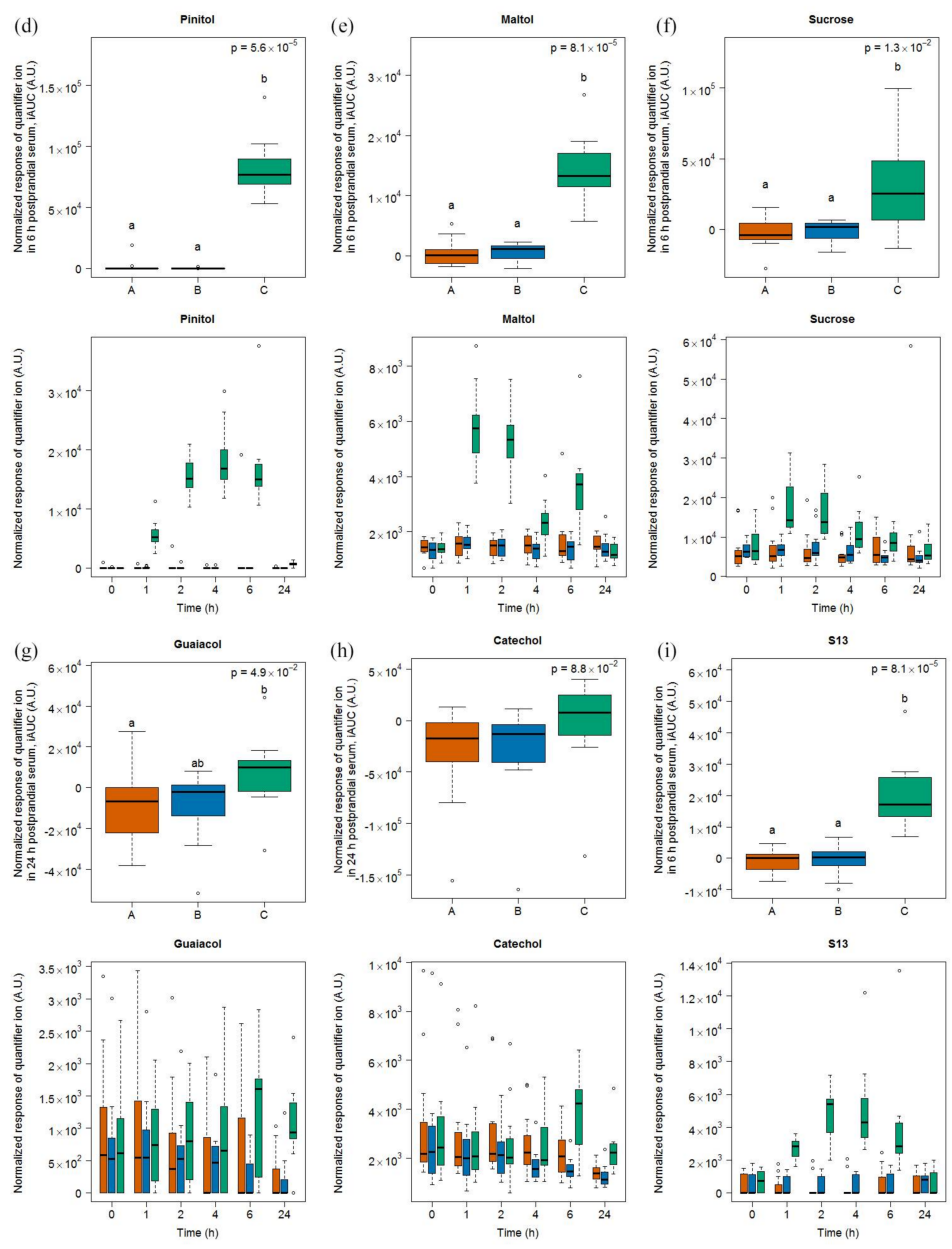

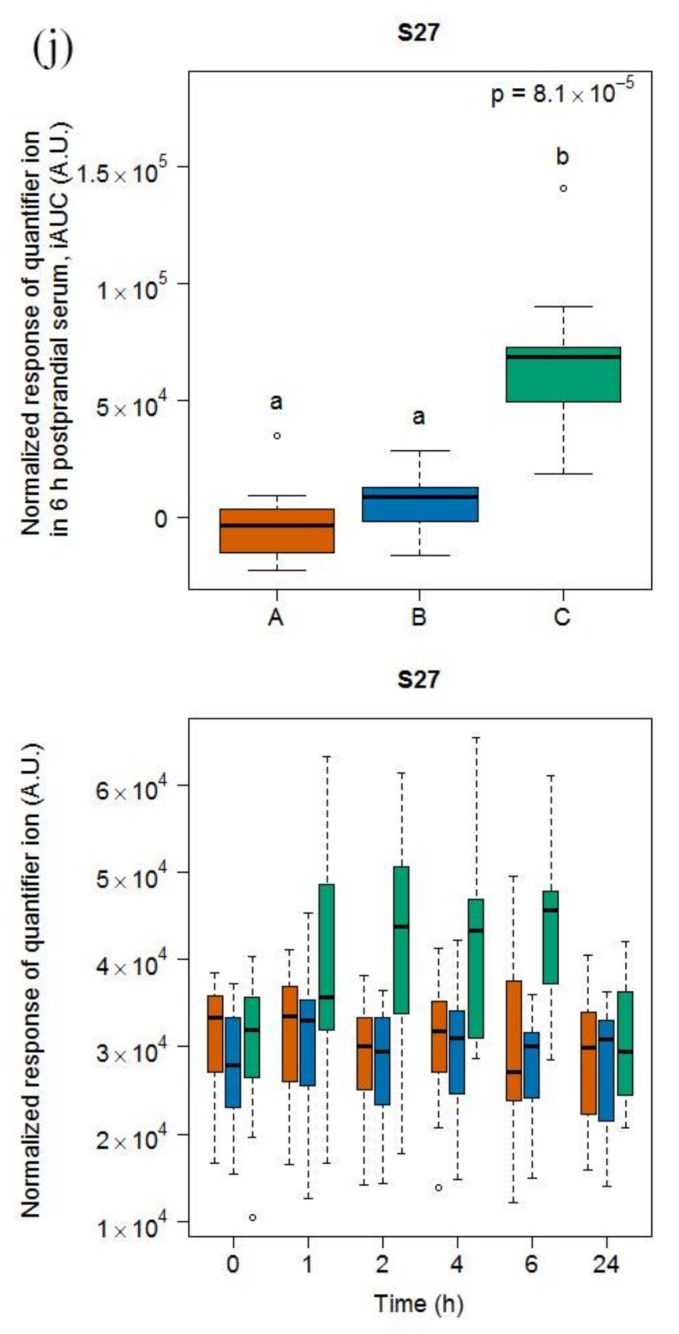
GC-MS-based postprandial behaviour of dodecanoic acid C12:0 (**a**), linoleic acid C18:2 (**b**), γ-tocopherol (**c**), pinitol (**d**), maltol (**e**), sucrose (**f**), guaiacol (**g**), catechol (**h**), unknown S13 (**i**), and unknown S27 (**j**) after soy drink intake (green) when compared to milk (orange) and cheese (blue) intake. Upper graph of panels: 6h-iAUC (catechol and guaiacol: 24h-iAUC); Lower graph of panels: time-points analysis from 0 to 24 h. The *p* values in the upper graphs are based on a Kruskal-Wallis test and have been adjusted for multiple testing. If the Kruskal-Wallis test was significant (*p* < 0.05), pairwise comparisons were conducted using a Conover-Inman test. Different letters (abc) denote significant differences based on a *p* value < 0.05.

Six compounds (dodecanoic acid, linoleic acid, γ-tocopherol, pinitol, and two unknown compounds S13 and S27) were discriminative for soy drink intake based on the OPLS-DA model comparing the 6h-iAUC after soy drink intake against milk and cheese intake (see Figure S5) and the subsequent targeted quantification (Figure 3). This list of discriminative compounds was complemented by the targeted analysis of four features, including maltol, sucrose, guaiacol, and catechol, already reported as discriminative for the soy drink in the urine samples of the same study [25]. Of note, all molecules showed a postprandial increase except linoleic acid, which showed a postprandial decrease. Among the four molecules targeted based on the literature, two of them (maltol, sucrose) showed significant increases in their 6h-iAUC after soy drink intake compared to milk and cheese intake. On the other hand guaiacol showed significant increases in its 24h-iAUC after soy drink intake compared to milk intake.

The serum levels of dodecanoic acid, pinitol, maltol, sucrose, and S13 were significantly higher after soy drink intake than after milk and cheese intake at 1, 2, and 4 h (see Tables S3 and S4). The serum levels of γ-tocopherol and S27 were significantly higher after soy drink intake than after milk and cheese intake at 4 and 6 h. Of note, despite apparent postprandial increases at late time points (i.e., 6 and 24 h), the serum levels of catechol and guaiacol were not discriminative at specific time points based on the Kruskal-Wallis sum rank test, although a trend was indicated by the significantly higher 6 h values of catechol after soy drink intake compared to cheese intake.

#### 2.1.4. Targeted Evaluation of C15:0 and C17:0 in Serum Samples

**Figure 4:**
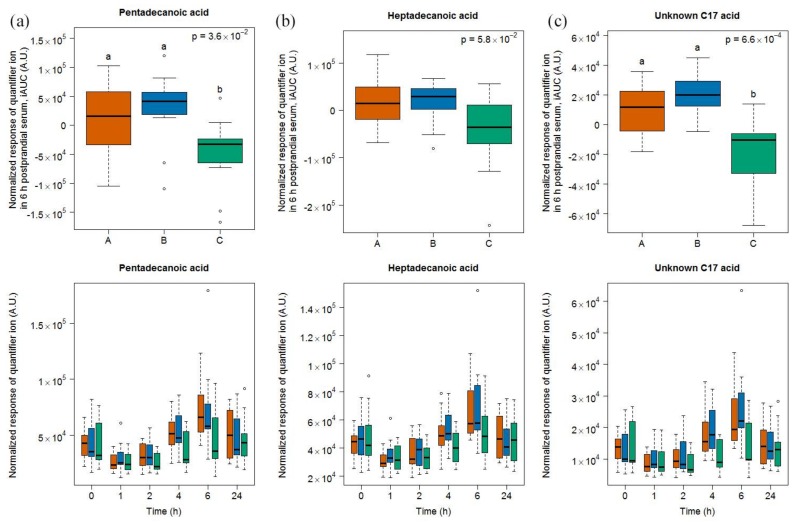
GC-MS-based postprandial behavior of pentadecanoic acid C15:0 (**a**), heptadecanoic acid C17:0 (**b**), and an unknown C17 acid (**c**), after milk (orange), cheese (blue), and soy drink (green) intake. Upper graph of panels: 6h-iAUC; Lower graph of panels: time-points analysis from 0 to 24 h. The *p* values in the upper graphs are based on a Kruskal-Wallis test and have been adjusted for multiple testing. If the Kruskal-Wallis test was significant (*p* < 0.05), pairwise comparisons were conducted using a Conover-Inman test. Different letters (abc) denote significant differences based on a *p* value < 0.05.

A targeted search for C15:0 and C17:0 revealed the presence of these two free fatty acids in the serum samples of our study. A significant difference between the 6h-iAUC was found for C15:0, but not for C17:0, based on a Kruskal-Wallis test of the three food groups. A Conover-Inman test for multiple comparison between milk and soy drink intake further revealed significant differences for C15:0, which were suggestive of a discriminating potential of this molecule (Figure 4). Interestingly, an unknown C17 acid was identified, by untargeted OPLSA-DA followed by univariate targeted analysis, as being discriminant of soy drink intake when compared to milk and cheese intake. The 6h-iAUC of this compound was indeed significantly higher after dairy intake than after intake of the soy drink.

After the intake of all the three foods, levels after 1 h were significantly lower when compared to baseline based on a paired Wilcoxon signed rank test, after milk intake (C15:0 *p* = 9.8 × 10^−4^, C17:0 *p* = 2.9 × 10^−3^, unknown C17 *p* = 1.9 × 10^−3^), after cheese intake (C15:0 *p* = 2.9 × 10^−3^, C17:0 *p* = 1.4 × 10^−2^, unknown C17 *p* = 1.4 × 10^−2^), and after soy drink intake (C15:0 *p* = 9.8 × 10^−4^, C17:0 *p* = 2.9 × 10^−3^, unknown C17 *p* = 9.8 × 10^−4^). The levels of C15:0, C17:0, and unknown C17 were then increased at 6 h compared to 0 h, after milk intake (C15:0 *p* = 9.8 × 10^−4^, C17:0 *p* = 2.0 × 10^−3^, unknown C17 *p* = 9.8 × 10^−4^) and cheese intake (C15:0 *p* = 2.0 × 10^−3^, C17:0 *p* = 2.0 × 10^−3^, unknown C17 *p* = 9.8 × 10^−4^) but not after soy drink intake (C15:0 *p* = 6.4 × 10^−1^, C17:0 *p* = 8.3 × 10^−1^, unknown C17 *p* = 6.4 × 10^−1^). A comparison of the postprandial response to the foods shows that the levels of C15:0, C17:0, and unknown C17 acid were higher 4 h after milk and cheese intake when compared to soy drink intake, and were higher 6 h after milk intake when compared to soy drink intake (see lower graph of panels in Figure 4 and Tables S3 and S4).

#### 2.1.5. Fatty Acid Amounts in Test Foods

**Figure 5:**
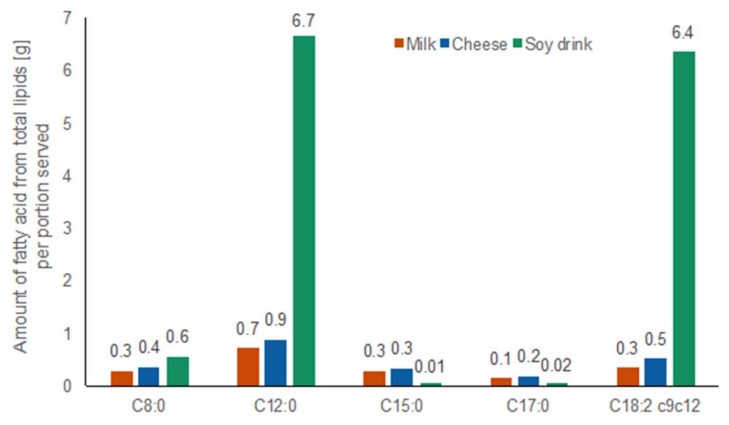
Amount of octanoic acid C8:0, dodecanoic acid C12:0, pentadecanoic acid C15:0, heptadecanoic acid C17:0, and linoleic acid C18:2 of total lipids in test foods per portion served measured by GC-FID. Milk: orange bars, cheese: blue bars, soy drink with added vegetable fat: green bars.

The amount of the fatty acids derived from total lipids that were relevant to this study in each test food are shown in Figure 5. The complete fatty acid profile of the three test foods is shown in Table S5. High resolution GC-FID analysis of fatty acid profiles of test foods revealed that total lipids of both dairy products were dominated by palmitic acid C16:0 (30% in milk, 26% in cheese), oleic acid C18:1 (16% in milk, 18% in cheese), myristic acid C14:0 (11% in milk, 26% in cheese), and stearic acid C18:0 (9% in milk, 10% in cheese). The total lipids of the soy drink, mostly derived from added vegetable fat composed of palm and palm kernel oil, consisted of the following four most abundant fatty acids: lauric acid C12:0 (22%), linoleic acid C18:2 (21%), palmitic acid C16:0 (13%), and stearic acid C18:0 (12.1%). The amounts of C15:0 and C17:0, the two dairy-specific odd-chain fatty acids, were low (0.3 g of C15:0 in 600 mL milk, 0.3 g of C15:0 in 100 g cheese; 0.1 g of C17:0 in 600 mL milk, 0.2 g of C17:0 in 100 g cheese).

### 2.2. NMR

The 6h-iAUCs of each of the 76 NMR bins were analyzed by univariate analysis (Kruskal-Wallis followed by Conover-Inman pairwise comparison) in 1 by 2 comparisons (milk vs. non-milk; cheese vs. non-cheese, soy drink vs. non-soy drink) to identify significant candidate markers for a specific food intake. Five spectral bins were significant and could be clearly assigned.

#### 2.2.1. Candidate FIBs for Milk

**Figure 6:**
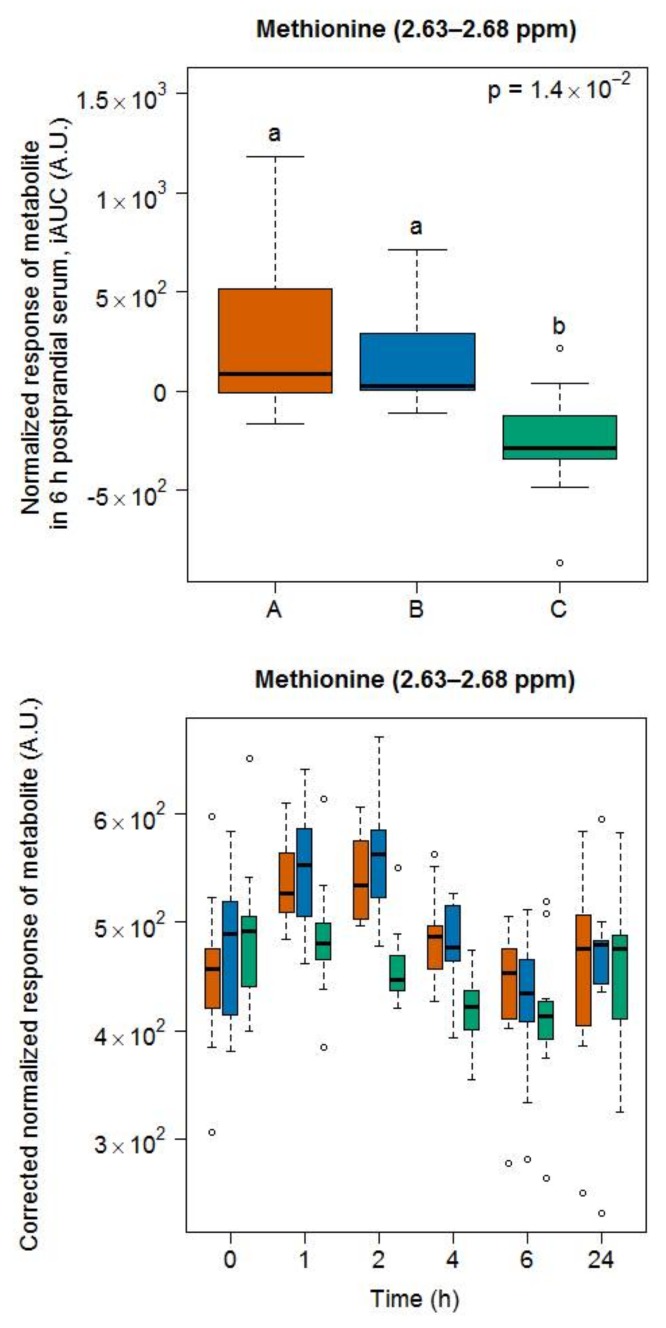
NMR-based postprandial behaviour of methionine after milk intake (orange) when compared to cheese (blue) and soy drink (green) intake. Upper graph of panels: 6h-iAUC; Lower graph of panels: time-points analysis from 0 to 24 h. The *p* values in the upper graphs are based on a Kruskal-Wallis test and have been adjusted for multiple testing. If the Kruskal-Wallis test was significant (*p* < 0.05), pairwise comparisons were conducted using a Conover-Inman test. Different letters (abc) denote significant differences based on a *p* value < 0.05.

A doublet at 2.63–2.68 ppm indicated that methionine was discriminative between dairy products (i.e., milk and cheese) and the soy drink (Figure 6). However, in contrast to GC-MS, which discriminated each of the three food groups (see Figure 2), NMR did not reveal a difference in the postprandial response of the subjects to milk and cheese intake.

#### 2.2.2. Candidate FIBs for Cheese

**Figure 7:**
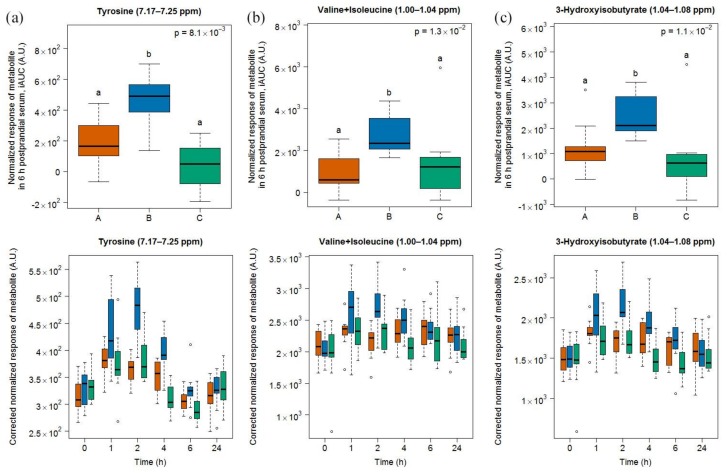
NMR-based postprandial behaviour of tyrosine (**a**), valine and isoleucine (**b**), and 3-hydroxyisobutyrate (**c**) after cheese intake (blue) when compared to milk (orange) and soy drink (green) intake. Upper graph of panels: 6h-iAUC; Lower graph of panels: time-points analysis from 0 to 24 h. The *p* values in the upper graphs are based on a Kruskal-Wallis test and have been adjusted for multiple testing. If the Kruskal-Wallis test was significant (*p* < 0.05), pairwise comparisons were conducted using a Conover-Inman test. Different letters (abc) denote significant differences based on a *p* value < 0.05.

Two doublets at 6.89–6.95 and 7.17–7.25 (Figure 7) ppm indicated an increased postprandial appearance of tyrosine after cheese intake, compared to milk and soy drink intake. Also, the region at 1.00–1.04 ppm, containing signals from the amino acids valine and isoleucine, had increased signals after cheese consumption, compared to milk and soy drink intake. Finally, the spectral region 1.04–1.08 ppm, containing a doublet originating from 3-hydroxyisobutyrate, also had increased signals after cheese intake, compared to milk and soy drink intake.

A kinetics analysis of the postprandial behaviour of these metabolites indicated that they reached maximal values at 1 or 2 h (lower graph in panels of Figure 7).

#### 2.2.3. Candidate FIBs for Soy Drink

**Figure 8:**
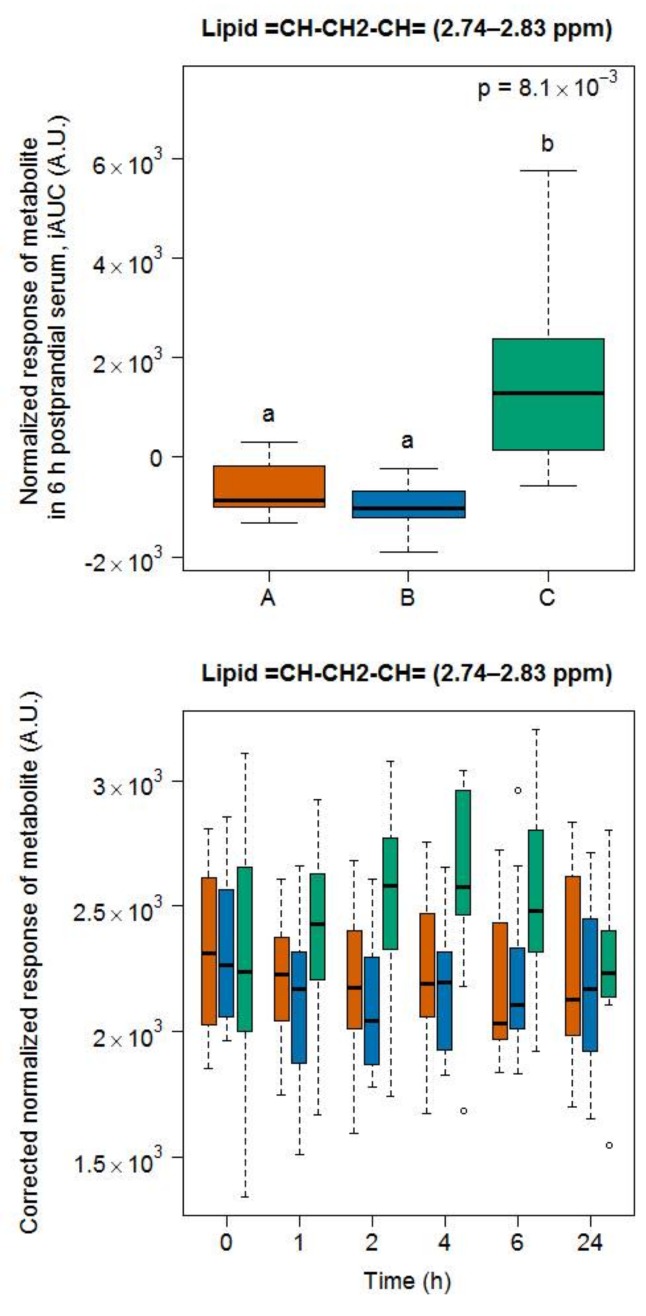
NMR-based postprandial behaviour of polyunsaturated lipid signals after soy drink intake (green) when compared to milk (orange) and cheese (blue) intake. Upper graph: 6h-iAUC; Lower graph: time-points analysis from 0 to 24 h. The *p* values in the upper graphs are based on a Kruskal-Wallis test and have been adjusted for multiple testing. If the Kruskal-Wallis test was significant (*p* < 0.05), pairwise comparisons were conducted using a Conover-Inman test. Different letters (abc) denote significant differences based on a *p* value < 0.05.

For soy drink, the region containing signals from polyunsaturated lipids (=CH–CH2–CH=) [26] was indicative of the intake of this test product, compared to the intake of milk and cheese (Figure 8).

An analysis of this region revealed slower postprandial kinetics than the other selected metabolites, reaching peak concentration around 4 h after intake and remaining at elevated levels at 6 h (lower graph in Figure 8).

### 2.3. Comparison of the Serum and Urinary Postprandial Profiles of Unmetabolized Candidate FIBs for Milk, Cheese, and Soy Drink

**Figure 9:**
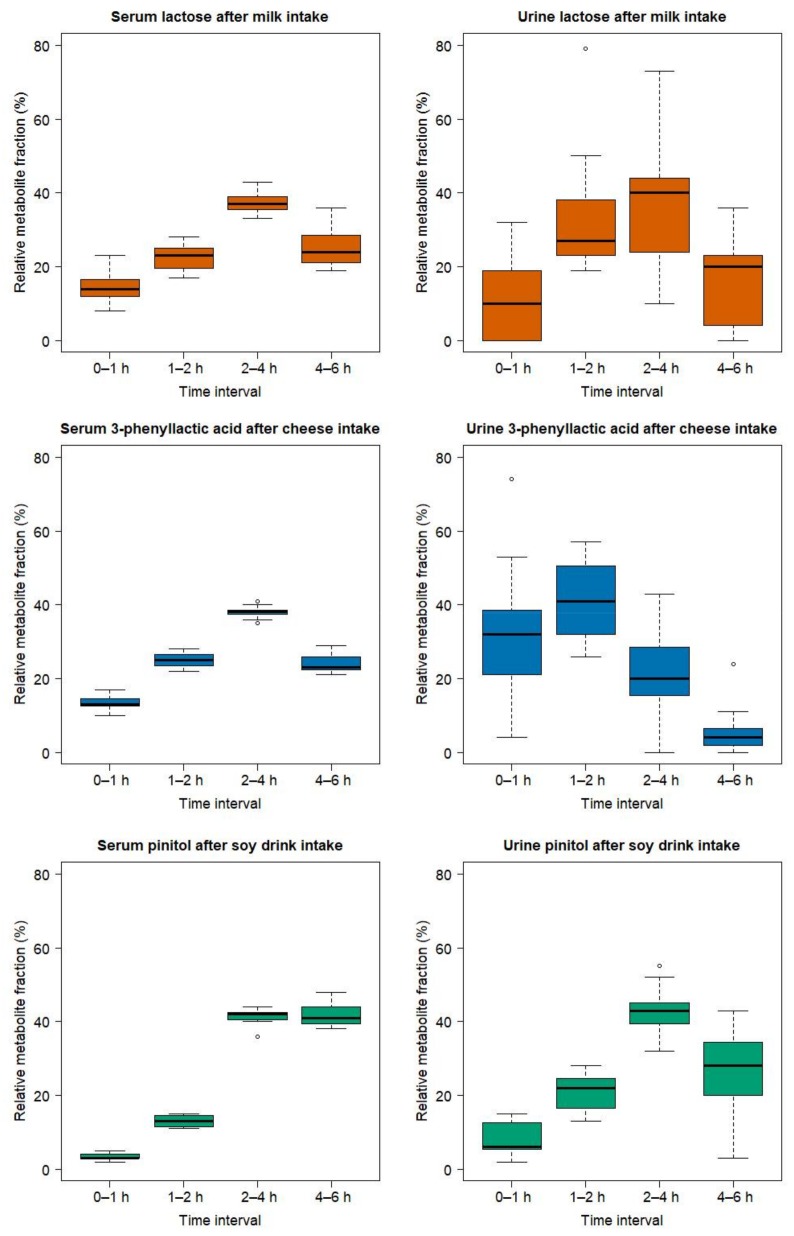
Boxplot of the GC-MS-based postprandial behaviour in serum (left panel) and urine (right panel) of lactose (orange), 3-phenyllactic acid (blue), and pinitol (green) after the ingestion of milk, cheese, and the soy drink, respectively. The x-axis represents time intervals (0–1 h, 1–2 h, 2–4 h, and 4–6 h). The y-axis represents, for each time interval, the fractional contribution in percent of each metabolite relative to the normalized cumulative response measured over the 6 h postprandial period. For serum, the fractional contribution of each time interval was calculated based on the 6h-AUC. For urine, the fractional contribution of each time interval was calculated using the normalized cumulative response, over the 6 h postprandial period, of the 4 urine pools (0–1 h, 1–2 h, 2–4 h, and 4–6 h) using the published data from the same subjects [25]. The untargeted urine measurements of lactose were characterized by a high technical variability, as measured by the CV of the QC samples [25]. In particular, lactose could not be detected in the urine samples of subjects 11 and 20, as well as in the QC samples of the batches co-analysed with these samples. The lactose values in urine in the middle right panel were consequently calculated by excluding subjects 11 and 20.

In order to visualize possible differences in the behaviour of unmetabolized candidate FIBs, the postprandial serum and urinary GC-MS profiles of lactose (after milk intake), 3-phenyllactic acid (after cheese intake), and pinitol (after soy drink intake) were visually compared (Figure 9). GC-MS data was used since all three metabolites were found in both biofluids by this platform. For each time interval (0–1 h, 1–2 h, 2–4 h, 4–6 h), the fractional contribution, in percent of each metabolite, is shown relative to the normalized cumulative response measured over the 6 h postprandial period. Whereas lactose (upper panels) and 3-phenyllalctic acid (middle panels) reached peak values in serum (left panels) in the 2–4 h time interval, pinitol values (lower panels) were still elevated during the 4–6 h time interval. The urine data (right panels), on the other hand, is characterized by a larger variability. A delayed expression of pinitol, compared to lactose and 3-phenyllactic acid, appears, however, to be evident during the 1–2 h interval. Of note, the AUCs values for serum are approximated using the trapezoid method, i.e., with linear interpolations between the time points 0, 1, 2, 4, and 6 h. As such, an exact comparison of the dynamic behaviour of each metabolite between serum and urine is limited.

## 3. Discussion

### 3.1. Stengtening the Evidence by Measuring Postprandial Kinetics of Candidate FIBs both in Serum and Urine by GC-MS and NMR

The validation of candidate FIBs is a multi-step process that requests the collection of a wide range of information. In that regard, we have used GC-MS and NMR metabolomics technologies to identify candidate FIBs for milk, cheese, and a soy drink (as a non-dairy alternative) as well as to characterize their specificities and postprandial kinetics in serum (this report) and in urine [25] using the same cohort.

A strength of this study is the emphasis given to the postprandial kinetics, which allowed us to get better insight into the absorption and metabolism of the candidate FIBs, thus providing more information to support their validation. In particular, a comparison of the postprandial kinetics of lactose after milk intake (peak value at 2 h), 3-phenyllactic acid after cheese intake (peak value at 2 h), and pinitol after soy intake (peak value at 4 h), under cross-over study conditions, provides interesting insights into the intestinal processing of these molecules. 3-phenyllactic and pinitol have been measured already in the test foods, hence they are not metabolized within the human body [25]. Also, if lactose is supplied in high concentration and/or if the subjects are lactose intolerant, this disaccharide is not fully metabolized and residual lactose can be absorbed and excreted intact [25,27], as will be discussed in more detail in Section 3.2. Indeed, although each of these molecules is directly transferred from their respective foods into the circulation, pinitol appears significantly later in the circulation compared to the two dairy markers. This observation raises interesting questions related to the relative contribution of the food structure, as well as of the intestinal absorption process, to this difference, which could be addressed by future research.

Furthermore, comparing the postprandial behaviour of these molecules in serum and urine reveals that, for all three metabolites, renal excretion in urine closely follows their appearance in serum. Similar findings were found for unmetabolized epigallocatechin after green-tea consumption [28].

Finally, the combined use of two analytical platforms (GC-MS and NMR) allowed us to identify candidate FIBs that were observed only with one of the two techniques, providing further evidence for the importance of a multi-platform investigation in nutrimetabonomic studies [25]. The flexibility provided by a broad analytical strategy was further demonstrated by the use of GC-MS to quantify the candidate FIBs in the foods ingested by the subjects. For illustration, we have taken a, as to yet unreported, targeted GC-MS approach that not only followed the postprandial fate of specific odd-chain fatty acids in serum but also measured them in dairy products. On the other hand, the discrepancy noted on the methionine results between the GC-MS and NMR results raises the issue of the analytical validation of metabolites derived from untargeted metabolomics methods as subsequent steps in the validation of candidate FIBs need to include harmonized sample preparation procedures as well as analytical methods whose performance (sensitivity, precision) are well characterized.

A more detailed discussion of the candidate FIBs identified for milk, cheese, and soy drink follows in the next three sections.

### 3.2. Lactose-Derived Metabolites as Candidate FIBs for Milk Intake

All of the candidate FIBs for milk intake identified by GC-MS in the serum samples (lactose, galactose, galactitol, galactonate, and galactono-1,5-lactone) have already been identified and discussed in details in the urine samples of the same study [25]. The identification of these compounds both in serum and urine strengthens their potential use as FIBs and motivates further research towards their validation.

Even though disaccharides were believed not to cross the intestinal brush border due to the absence of an active transport system, lactose has previously been detected after the intake of a dairy shake in blood [29] and, more recently, after the intake of milk and even yoghurt [27,30]. Interestingly, the lactose levels are almost back to their baseline level at 6 h in our study in which the subjects have ingested 600 mL of milk, whereas significant amounts of lactose remained in serum 6 h after the ingestion of 800 mL [27]. This difference, which may be due to limitation in the passive absorption properties of the intestinal barrier, highlights the importance of dose-response studies in the field of food intake markers.

Galactose derives from hydrolysis of lactose during digestion and was rapidly absorbed and metabolized since it was no longer detectable in serum after 2 h. Under normal conditions, galactose enters the Leloir pathway and is metabolized to UDP-glucose. In case of excess galactose, free galactose also enters two additional pathways, being either reduced to galactitol or oxidised to galactonate via galactono-1,5-lactone [31,32,33]. These metabolites are indicative of milk intake in serum in our study in which the volunteers consumed 600 mL of milk within 15 min. As already discussed in Münger et al. [25] it remains uncertain whether these markers will appear postprandially when the amounts of milk consumed are decreased to nutritionally-relevant levels. However, under our study conditions, galactonate and galactose were detected postprandially after milk intake but not under fasting conditions, whereas lactose, galactitol, and galactono-1,5-lactone were also measured at detectable concentrations in fasting sera (0 and 24 h samples). This could indicate that lactose is found in serum as a marker of chronic consumption of lactose-containing products, since all the recruited subjects are dairy consumers. We hypothesize that the fasting levels of these molecules may result from their accumulation, via an unknown mechanism, after regular intake of milk or dairy products containing lactose, which makes them attractive candidate FBIs for the intake of lactose-containing products. In that regard, Playdon et al. [34] recently proposed galactonate as a marker for dairy product intake. However, the absence of galactonate after cheese intake and its presence after milk intake in our study, indicates that galactonate should rather be regarded as a candidate urinary and serum FIB for dairy products containing lactose.

In the 6h pooled postprandial urine samples, lactose, galactose, and galactonate were among the main metabolites detected by NMR as specifically increased after milk intake [25]. In serum, these molecules were found by GC-MS after milk intake but their low concentrations in blood, together with their fast absorption and excretion kinetics, prevented their detection by NMR, this technique being less sensitive compared to GC-MS.

### 3.3. Amino Acid-Derived Metabolites as Candidate FIBs for Cheese

Most of the metabolites related to cheese intake were reflective of protein fermentative and/or digestive metabolism. GC-MS revealed that methionine, proline, and leucine had increased postprandial levels after cheese intake compared to the other two products. NMR found the same phenomenon for tyrosine, valine, and isoleucine, however, not for methionine. In support to these findings, free amino acids were also the most predominant metabolites identified after cheese intake in urine samples of the same study [25]. Although amino acids are obviously not suitable as FIBs because they are ubiquitously present in foods and released during digestion, their increased postprandial appearance after cheese intake, compared to milk, is interesting as it might reflect the ability of the milk fermentation process to further increase the release of free amino acids for their uptake into the circulation:

- A survey of the free amino acids released during the ripening of a range of Swiss cheese showed that proline was among the most released amino acids in Gruyère [35]. Postprandial proline appeared in the plasma of healthy adults after ingestion of whey proteins [36] and in infants fed a milk-based formula [37].
- Methionine was among the most released amino acids during the ripening of Appenzeller cheese [35]. Postprandial methionine levels were increased in infants fed a milk-based formula [37] as well as in the plasma of obese subjects fed a whey isolate [38] but not as much after the intake by obese subjects of a whey hydrolysate [39], probably due to oxidation into its sulfoxide in the latter product. Of note, as whey is removed during the production of Gruyère cheese, the high content of methionine in casein could still explain our finding. In that context, it is interesting to note that Stanstrup et al. [38] reported similar postprandial kinetics than ours, although casein and whey proteins are digested with different kinetics. The cheese ripening process is, however, likely to eliminate these differences as a consequence of the pre-digestive properties associated with the fermentation process.
- Leucine was among the most released amino acids during the ripening of Gruyère [35]. The iAUC of this amino acid could be increased in infants fed a milk-based formula [37], in a dose-dependent manner in healthy adults fed whey protein [36], and in healthy older people by supplementing a whey protein extract with leucine [40].
- Glutamic acid increased upon ripening of a model cheese [41] as well as in Gruyère [35]. Glutamic acid also increased postprandially in humans after the intake of whey proteins, although not in a dose-dependent manner [36].
- Tyrosine is also one predominant amino acid in casein [42]. The concentration of tyrosine was shown to increase during cheese ripening in a model cheese [43] as well as in Gruyère [35]. This amino acid was also found in higher concentration in urine when cheese was consumed, both in comparison with a control meal and with milk [23]. Postprandial tyrosine also appeared in a dose-dependent manner in the plasma of healthy adults after ingestion of whey proteins [36] and in infants fed a milk-based formula [37].
- Valine and isoleucine are two of the main amino acids in casein [42] and their concentrations increase during ripening [35]. The iAUC of these two amino acids could be increased in infants fed a milk-based formula [37], in a dose-dependent manner in healthy adults fed whey protein [36], and in healthy older people by supplementing a whey protein extract with leucine [40]. Both amino acids were also increased in the plasma of obese subjects after the intake of a whey isolate [39] as well as after intake of a caseinoglycomacropeptide [38], a whey protein derivate rich in these two amino acids. These insulinotropic amino acids are rapidly taken up by the organism peaking postprandially at 1 to 2 h [38,44,45], as also reported in our results. Interestingly, 3-hydroxyisobutyrate was found by NMR in higher concentrations in the serum of our subjects after cheese intake. The postprandial concentration of this organic acid, which is an intermediate in the catabolism of valine, was also increased in infants fed a milk-based formula [37].

Despite their lack of attractiveness as candidate FIBs, the increased postprandial appearance of amino acids observed in the serum of the subjects after cheese intake deserves attention in light of the relevance of dietary protein and amino acid supplementation to improve muscle mass and strength in older adults [46]. In particular, dairy proteins, because of their high content in key amino acids such as leucine, are well recognized dietary interventions for sarcopenia [47]. In analogy to a similar observation made while comparing the postprandial response of human subjects to milk and yoghurt intake [30], our results suggest that the fermentation of milk to cheese increases the bioavailability of dairy amino acids, as shown for proline, leucine, glutamic acid, tyrosine, valine, leucine, and eventually methionine, what makes milk fermentation an attractive approach for the management of muscle metabolism.

In addition to the pre-digestive properties of food fermentation, the bacterial transformation of free amino acid by lactic acid bacteria provides innovative solutions for the identification of candidate FIBs for food fermentation. In that regard, 3-phenyllactic acid, a bacterial product of phenylalanine fermentation in dairy products, was exclusively detected after cheese ingestion in the urine [25] and serum samples of this study. As the baseline levels in serum and urine were below the limit of detection, 3-phenyllactic acid may be a suitable FIB for fermented foods, during the first 6 h after intake as it is then excreted non-metabolized into urine [25].

### 3.4. Heterogeneous Molecular Pattern of Candidate FIBs for Soy Drink

In addition to the results obtained in serum, pinitol, maltol, sucrose, guaiacol, and catechol were also indicative of the soy drink intake in urine [25]. Pinitol seems, however, to be the only specific candidate FIB for soy drink intake as it was neither present in serum at baseline, all other compounds being also at detectable levels at baseline, nor after the intake of milk and cheese. Pinitol is a cyclic polyol predominantly occurring in the *Fabacea* plant family [48].

Interestingly, serum γ-tocopherol was also discriminative of soy drink intake. γ-Tocopherol is a major vitamin E form and its serum levels in humans are negatively correlated with triglycerides and total cholesterol consumption [49]. γ-Tocopherol is present, although not exclusively, in soy products including soybean oil [50] and soybean seeds [51] and postprandial increases in serum γ-tocopherol have been reported after intake of corn and sesame oil [52]. Of note, γ-tocopherol was not identified in urine in our study [25] in agreement with its metabolism and excretion in the form of carboxyethyl hydroxychromanols [52].

In urine, the metabolites with a larger postprandial excretion after soy intake, compared to milk and cheese, were pyridoxine, trans-aconitate, and trigonelline [25]. These molecules could not be detected in the serum spectra, probably due to lower concentrations in this biofluid as well as signal overlap with the lipid-like molecules present in high amounts in serum.

Linoleic was enriched in the soy drink following the addition of vegetable fat. In agreement with the GC-MS data on linoleic acid (see Section 3.5), the NMR region from 2.74 to 2.83 ppm, containing signals assigned to polyunsaturated lipid “=CH–CH2–CH=” [26], increased between 4 and 6 h after intake of the soy drink.

### 3.5. Targeted Lipid Analysis in Food and Serum

To our knowledge, only two intervention studies have so far investigated the link between dairy fat intake and blood levels of odd-chain fatty acids [18,19]. In contrast to our study, the subjects were investigated under free-living conditions and the dietary intervention lasted for several weeks. In both studies, the amounts of C15:0 and C17:0 in blood correlated positively with the amount of dairy fat consumed. These outcomes were substantiated by data from observational studies showing that blood levels of odd-chain fatty acids positively correlate with the consumption of dairy fat [14,15,16,17]. Importantly, the occurrence of odd-chain fatty acids in serum is not only attributed to dairy intake but also to fish intake [53] and their role as dairy fat biomarkers is questioned. Novel findings also show that C17:0 is synthesized in vivo and would therefore not be a suitable FIB [21]. Furthermore, the biosynthesis may be decreased by other dietary compounds leading to constant levels of C17:0 in blood even though dairy fat is ingested [21].

Our data demonstrates that, at baseline, both odd-chain fatty acids, as well as an unknown C17 acid, were at detectable levels in the serum of the volunteers, who were regular consumers of dairy products even though they were not allowed to consume dairy products in the 48 h of the run-in phase of this study. In order to obtain isocaloric test foods by roughly achieving comparable macronutrient composition between the three test foods, vegetable fat was added to the soy drink. The 6h-iAUCs of C15:0 and the unknown C17 acid were higher after dairy intake than after soy drink intake. Intriguingly, C15:0, C17:0, and the unknown C17 acid showed a postprandial decrease until 4 h after the intake independent from the food ingested. Whereas this postprandial kinetic behaviour of C15:0 has not been reported yet, Pellis et al. [29] reported findings similar to ours for C17:0 with a postprandial increase in the plasma concentrations of this fatty acid after a lag time of 4 h in response to the intake of a dairy shake. Interestingly, in our study, the postprandial serum concentrations of C15:0, C17:0, and the unknown C17 acid were higher 4 h after milk and cheese intake when compared to soy drink intake, and were higher 6 h after milk intake when compared to soy drink intake. Taken together, our data indicates that dairy fat has an impact on the levels of C15 and C17 odd chains fatty acids.

In agreement with their metabolic fate, several fatty acids with an even number of carbon atoms (C8:0, C12:0, and C18:2) were detected postprandially after the intake of the soy drink in the serum (this report) but not in urine samples [25]. Of note, the free fatty acids detected in serum do not derive from the soy product itself but from the vegetable fat that was added in order to obtain isocaloric test foods. As shown by the fatty acid profiles of total lipids of the test foods, C12:0 was the dominant fatty acid in the soy drink containing vegetable fat composed of palm and palm kernel oil. Interestingly, the medium chain fatty acid, C12:0, which is known to be a poor substrate for re-esterification into triglycerides before systemic transport [29], showed a postprandial increase between 0 and 6 h in the sera. This increase may be attributed to a direct release of this molecule into the blood without re-esterification. The long-chain fatty acid, C18:2, known to be re-esterified after absorption, showed an initial postprandial decrease and only increased 6 h after soy drink intake, which supplied the highest amount of C18:2 among the three foods. The postprandial decrease could be attributed to inhibition of lipase by insulin in the postprandial state [29].

### 3.6. Limitations of Study

The specificity of the candidate FIBs reported in this study need to be evaluated in a broader dietary context. For illustration, 3-phenyllactic acid is a natural broad-spectrum antimicrobial compound widely existing in honey and fermented foods, which can be produced by many microorganisms, especially lactic acid bacteria [54]. As such, 3-phenyllactic acid was not only identified after cheese intake (this report) but also after the intake of yoghurt [30]. Consequently, this molecule is more likely to be a marker for the intake of fermented foods rather than cheese. On the other hand, the candidate FIBs found in our study could have a narrow specificity for the selected food products. This issue is particularly relevant for products with a broad spectrum of technological transformation, e.g., cheese. The validity of these FIBs for the ingestion of other types of cheese should thus be investigated [55].

The limited number of foods, which can be investigated for candidate FIBs in a single intervention study can be overcome by concerted research programs. This strategy is followed by the FoodBAll consortium, which is currently investigating fourteen foods, covering the most important food groups, in seven intervention studies. A collective and harmonized analysis of the candidate FIBs derived from these studies will broaden the knowledge of the specificity of these markers [10].

Not all serum metabolites identified as candidate FIBs in this report were also found in the urine samples of the same study [25]. To a large extend this discrepancy is obviously related to the intrinsic relationship of the two biofluids with respect to the metabolic fate of the absorbed nutrients and the excretion of the resulting metabolites, highlighting the relevance of investigating both biofluids.

Unusually large amounts of the test foods were given to the subjects to enable a more secure identification of candidate FIBs. The relevance of the candidate FIBs identified under these conditions should be addressed by measuring them in biofluids of subjects under free-living conditions.

Most of the selected metabolites displayed fast kinetics, most of their serum postprandial levels having returned to baseline values at 6 h. It will thus be necessary to determine whether the most promising candidate FIBs accumulate under fasting conditions in biofluids due to the regular consumption of the corresponding foods.

Finally, a significant inter-individual variability is observed in the postprandial behaviour of the candidate FIBs, its extent being different from one metabolite to the other as illustrated for the unmetabolized lactose, 3-phenylalanine, and pinitol. The variance of these metabolites in serum depends on the capability of the gastro-intestinal tract of each subject to release these molecules from the food matrix and to absorb them across the gut barrier. The variance in urine further derives from the efficiency of renal filtration and excretion. Characterizing the inter-individual variability of candidate FIBs, however, requests that the analytical variability is known. This information is usually not available from the design of studies such as ours, which take an untargeted strategy. In that context, a study investigating the inter-individual variation in the plasma uptake of the unmetabolized α-tocoherol has shown differences of up to 40-fold [56]. The importance of understanding the inter-individual variability in absorption, metabolism, and excretion of food metabolites is key, especially for the metabolites for which health benefits are claimed [57].

In addition to providing several candidate FIBs for the intake of dairy and soy products, our study suggests an attractive workflow for FIBs discovery, which might comprise (a) blood analyses to provide mechanistic information on the metabolism of candidate FIBs of value and (b) their validation in urine samples that can be collected less invasively than blood.

## 4. Materials and Methods

### 4.1. Study Design

This randomized, controlled crossover study has been performed within the frame of the European JPI-funded project Food Biomarker Alliance (FoodBAll). The present study was conducted according to the guidelines laid down in the Declaration of Helsinki and was approved by the Ethical Committee of Canton Vaud, Switzerland. The trial was registered at clinicaltrials.gov (NCT02705560). All volunteers signed an informed consent form. All details on recruiting procedure, inclusion criteria, characteristics of study population, study design, test product, and standardized meal compositions have been published elsewhere [25]. In short, eleven healthy volunteers (5 women and 6 men) between the age of 19 and 31, with a normal BMI (>18 and <30 kg/m^2^) and regularly consuming dairy products have been included. The number of subjects considered, even though low, was sufficient to identify candidate FIBs in urine [25] and was selected following a common protocol used by the FoodBAll consortium [10].

**Figure 10:**
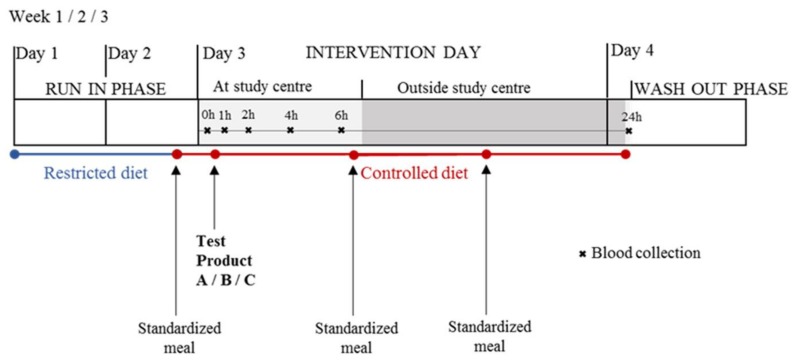
Schematic representation of the randomized, controlled, cross-over study with three test products (A = milk, B = cheese, C = soy drink as a comparison). Serum samples were collected after at least 12 h of fasting at baseline and 1, 2, 4, 6 and 24 h after the consumption of the test product. Volunteers followed a 2-day restricted diet and consumed a standardized meal for dinner during the run-in phase. During the day of intervention and up to 24 h after the ingestion of the test product, the diet was fully controlled.

During a 2-day run-in phase, the volunteers followed a restricted diet (no dairy products, no fermented products, no soy products, and no products of bovine origin) and, during the intervention phase up to 24 h, the diet was fully controlled by providing three times a standardized meal apart from the test product [25] as shown in Figure 10.

After at least 12 h of fasting, blood samples were collected at the study centre before (t = 0 h) and 1, 2, 4, and 6 h after the ingestion of the test product using an indwelling peripheral venous catheter. The order of test products for each volunteer was randomly assigned. Randomization was performed using the rand function in Excel by allocating the 6 possible test product sequences among the male volunteers and among the female volunteers so that each sequence appeared only once in each gender group. The test products were 600 mL organic pasteurized full fat milk (3.9% fat), 100 g hard cheese (Le Gruyère AOP, Bulle, Switzerland) along with 500 mL of water, and 600 mL soy drink, which was a mixture of soy drink and added vegetable fat. All three test products were isocaloric (400 kcal). At lunch, 6h after the consumption of the test product, participants consumed a standardized meal at the study centre. Then later, for dinner, they consumed the same standardized meal in free-living conditions. Twenty-four hours after the ingestion of the test product, again in fasted state, the volunteers returned to the centre and another blood sample was collected. Blood samples were collected in 4.7 mL vacutainer tubes (S-Monovette^®^-Sarstedt, Nümbrecht, Germany), were incubated for 30 min at room temperature to allow clotting, and centrifuged at 1800 g for 10 min at 4 °C. Supernatant was aliquoted into smaller volumes and stored at −20 °C overnight before being transferred to −80 °C for long-term storage.

### 4.2. Untargeted GC-MS Analysis of Serum Samples

The sample preparation was based on Dunn et al. [58] with some modifications. In short, 50 µL of an internal standard solution (labelled D-fructose, U-13C6, 99%, Cambridge Isotope Laboratories, Inc., Cambridge, UK) was added to serum samples (100 µL), which was followed by precipitation with 300 µL cold methanol, centrifugation, transfer of supernatant (370 µL), and drying using a vacuum centrifuge. The samples further underwent a two-step derivatization and were subjected to analysis on a GC-MS 7890B/MS5977A (Agilent Technologies, Santa Clara, CA, USA) with a CombiPAL autosampler (CTC-Analytics AG, Zwingen, Switzerland) and a DB-5 ms fused silica capillary column (60 m, 0.25 mm i.d., 0.25 μm film thickness, Agilent Technologies, Basel, Switzerland). The samples were injected into a multimode injector (MMI) using the following temperature program: initially 90 °C, heating rate 900 °C/min until 280 °C, cooled for 5 min at rate of 30 °C/min, and kept at 250 °C. The oven program was as follows: initial temperature 70 °C for 2 min, increase up to 160 °C at a rate of 5 °C/min, increase to 300 °C at a rate of 10 °C/min, which was held for 16 min, equilibration time 1 min. MS detection mass ranged from 28.5 to 600 Da, MS source temperature was 230 °C, and MS Quad temperature was 150 °C. Electron ionisation was performed with 70 eV.

One batch consisted of all samples (*n* = 18) of one volunteer. The order of the samples within one batch and the order of volunteers were randomized using the rand function in Excel. Quality control samples (QC) were prepared beforehand by mixing all serum samples at equal volumes. Each batch was initiated by five injections of QC samples for equilibration and after every 5th serum sample a fresh QC was injected. At start and end of one batch, a blank sample (pure water) was included. QC samples and blank samples underwent the same sample preparation as serum samples. The deconvolution within the acquired chromatograms was performed using eRah package [59] (version 1.05) in R environment (settings: min peak width = 2 s, max peak distance = 5 s, min.spectra.corr = 0.85, avoid.processing.mz = c(28.5:69,73:75,147:149), mz.range = 70:600, missing compound recovery step = 5). In the resulting list containing the deconvoluted features, features with retention time before 10 min were removed (reagents region).

The untargeted GC-MS analysis, pre-processing of data as well as statistical analyses have been performed on serum samples as reported for the urine specimens of this study [25]. A non-parametric test for longitudinal data was performed on the deconvoluted dataset in R (R V3.1.2, nparLD package) [60] in order to select features presenting a significant postprandial time response after the intake of one of the three foods (*p* < 0.05 for time effect as a cut-off for significance). The incremental area under the curve for the 6h postprandial phase after the intake of each food (6h-iAUC) was calculated for each feature in R (MESS package) [30]. The resulting dataset was then UV scaled and subjected to multivariate data analysis in SIMCA-P software (V.14.0, Umetrics, Umea, Sweden). Principal Component Analysis (PCA) was used for detection of outliers (all samples being inside the Hotelling’s T^2^ ellipse using 0.01 as a confidence level), PLS-DA for classes separation (when more than two classes were compared), and OPLS-DA for classes separation when applying 1 by 2 comparisons (milk vs. cheese/soy drink, cheese vs. milk/soy drink, and soy drink vs. cheese/milk). For selection of discriminative compounds, features with VIP values higher than 2 were selected, which was also based on a visual analysis of the loading plot. Only models with predictive ability parameter Q2 > 0.5 were considered. In certain cases, deconvolution of chromatographic peaks resulted in several features even though the peak was deriving from a single compound. In these cases, features with identical retention time (RT) and identical characteristic ions were regarded as one compound. The identification of compounds was based on an in-house reference compound library using retention index (RI) and spectral data. For the selected discriminating compounds (VIP > 2), quantifier and qualifier ions were retrieved from spectral data after deconvolution. Using these ions, a targeted evaluation of these compounds was performed in MassHunter Quantitative Analysis (Agilent Technologies, Santa Clara, CA, USA) in order to optimize integration in each individual chromatogram. In case of stereoisomeric forms of selected discriminating features formed during derivatisation (e.g., in case of lactose), the peak with the highest response was further evaluated. Based on the responses of the quantifier ions normalized with quantifier ion of the internal standard, the 6h-iAUC was calculated. Univariate analysis using Kruskal-Wallis test was performed in R and, if differences between the iAUC attributed to the intake of the three foods were significant, a Conover-Inman test (Conover package) for multiple comparison was applied to compare the postprandial responses based on iAUC after intake of the three foods. *p* values were adjusted using the Benjamini Hotchberg correction for multiple testing, with adjusted *p* < 0.05 as a cut-off for significance. If the 6h-iAUC of one test foods was significantly different from the other two test foods, the compound was regarded as a candidate FIB. Kinetics of candidate FIBs were assessed based on normalized responses of quantifier ion using targeted data evaluation approach in Masshunter Quant. Differences at each time-point between the foods were assessed with the Kruskal-Wallis test and multiple pairwise comparison was carried out using the Conover-Inman test based on delta values (subtraction of baseline value). In addition, compounds found to be indicative of either milk, cheese, or soy drink intake in urine as published in [18,19] but that were not identified as markers based on multivariate analysis in serum, were directly assessed in the serum data using the targeted approach with the quantifier ions, in order to validate previous results.

### 4.3. Assessment of Serum Pentadecanoic and Heptadecanoic Acid by GC-MS

Pentadecanoic acid (C15:0) and heptadecanoic acid (C17:0) were evaluated in the GC-MS data in a targeted approach by assessing the response of their quantifier ions at known retention time normalised with the quantifier ion of internal standard. The GC-MS method used here allows only to detect fatty acids in their free form as no prior re-esterification step is included in the sample preparation. The iAUC was calculated until 6 h (6h-iAUC) and univariate analysis using Kruskal-Wallis rank test was performed to detect differences between foods. Furthermore, to identify a postprandial effect for each food, values obtained at each time point were compared to baseline using the paired Wilcoxon’s signed rank test. As for the untargeted analysis above, differences at each time-point between the foods were assessed with the Kruskal-Wallis test and multiple pairwise comparison was carried out using the Conover-Inman test based on delta values (subtraction of baseline value).

### 4.4. Fatty Acid Profiles of Total Lipids of Test Foods Assessed by High Resolution GC FID

In addition to the already published macronutrient composition of the test products [25], the fatty acid profile of total lipids was assessed by targeted high resolution GC FID method. The lipids were extracted from milk and soy drink samples after an ammonia-alkaline digestion at room temperature with a mixture of diethyl ether and n-pentane. Cheese samples were extracted with n-pentane in a Soxhlet apparatus. The purified fat was recovered from the extracts by evaporation of the solvent. After dissolution of 300 mg of the purified fat in n-hexane (containing nonanoic acid as internal standard), the glycerides were trans-esterified and the free fatty acids were esterified to the corresponding methyl esters by a solution of potassium hydroxide in methanol. All analyses were performed according to Collomb and Bühler [61] on an Agilent model 6890 gas chromatograph with an on-column injector and a flame ionisation detector (Agilent Technologies, Basel, Switzerland). The fatty acid methyl esters were separated on a CP-Sil 88 column (100 m, 0.25 mm i.d., 0.20 µm film thickness; Varian BV, Middleburg, The Netherlands) and quantified using the ChemStation software package B.04.02 (Agilent Technologies, Basel, Switzerland).

### 4.5. NMR Sample Preparation and Analysis

Serum aliquots were stored at −80 °C until their use for the NMR analysis. A serum sample of 500 μL was placed in a clean microfuge tube containing 130 μL of D_2_O-based phosphate buffer pH 7.4 containing also 20 mM 4,4-dimethyl-4-silapentane-1-sulfonic acid (DSS-d_6_) as chemical shift reference standard, 70 mM sodium azide (NaN_3_) as an antibacterial agent [62] and 20 mM 2-chloro pyrimidine-5-carboxylic acid (2CLPYR5CA) as reference standard. The inclusion of 2CLPYR5CA provides a down field signal that greatly improves phasing result during automatic spectral processing. Poorly phased spectra give rise to quantification errors due to erroneous area calculations under each peak. 2CLPYR5CA is stable at room temperature for more than 1 week and its signal resonates at 8.773 ppm at pH 7.40, a frequency which does not overlap with the signals of the biological metabolites so far identified in serum. The mixture was homogenized by vortexing for 30 s and 590 μL were transferred into 5 × 178 mm (7″) 5 mm outer diameter NMR tubes (for Bruker Match holder). ^1^H-NMR spectra were recorded at 298 K with an AVANCE spectrometer (Bruker BioSpin, Karlsruhe, Germany) operating at a proton frequency of 600.13 MHz, equipped with an autosampler with 60 holders. A standard spin echo Carr–Purcell–Meiboom–Gill (CPMG; cpmgpr1d.comp; Bruker BioSpin, Karlsruhe, Germany) pulse sequence with 256 scans (NS), 32768 data points (TD), a spectral width (SW) of 11.9705 ppm, an acquisition time (AQ) of 2.28 s, and a saturation time of 0.3 milliseconds (D20). A relaxation delay (D1) of 4 s was needed to reduce the signals arising from macromolecules. The data were Fourier transformed and phase and baseline corrections were automatically applied using TopSpin version 3.0 (Bruker BioSpin, Karlsruhe, Germany). Signals were assigned by comparing their chemical shift and multiplicity with Chenomx software data bank (version 8.1, Edmonton, AB, Canada).

### 4.6. Analysis of NMR Spectra

Serum NMR spectra were exported in ASCII file format. The ASCII files were then imported into R software (version 3.3.2) for pre-processing and later statistical analysis. Firstly, chemical shift referencing was performed by setting the reference signal of DSS to 0.00 ppm. The uninformative spectral regions, displaying only noise (i.e., spectral peripheral regions below 0.5 ppm and above 10 ppm), and the region which is strongly affected by the residual solvent signal (between 4.7 and 4.95 ppm) were removed prior to data analysis. Normalization was carried out using the PQN algorithm [63]. For each subject, the spectra from the following time-points were pooled for statistical analysis: 1, 2, 4, and 6 h. The pooled spectral matrices were then imported in MatLab (R2014b, MathWorks Inc., Natick, MA, USA). In total 76 signals, significantly above the baseline, a S/N ratio >3 being used to define the limit of detection [64,65]. Postprandial 6h-iAUCs after the intake of milk, cheese, or soy were compared through univariate statistical analysis using the Kruskal-Wallis test and the Conover-Inman test for pairwise comparison. *p* values were adjusted for multiple testing using the Benjamini Hochberg correction (adjusted *p* < 0.05 as a cutoff for significance).

## Supplementary Materials

The following are available online at http://www.mdpi.com/2218-1989/8/2/26/s1. Figure S1: PLS-DA of postprandial serum samples using 6 h incremental area under the curve, with foods as classes (milk intake, cheese intake, and soy drink intake) assessed by GC-MS. Score plot (a), permutation test (b) and loading plot (c). Discriminant features (VIP > 2) are indicated in red on the loading plot, with indication regarding their identities. Figure S2: PLS-DA score plot of serum samples after milk intake, cheese intake, and soy drink intake assessed by GC-MS, with time as classes. Median of the 11 subjects was used for each time point and each food. Figure S3: OPLS-DA of postprandial serum samples using 6 h incremental area under the curve, assessed by GC-MS, with two classes for feature selection: samples after milk intake (class 1) and cheese/soy drink intake (class 2). Score plot (a), permutation test (b) and loading plot (c). Discriminating features (VIP > 2) are indicated in red on the loading plot, with indication regarding their identities. Figure S4: OPLS-DA of postprandial serum samples using 6 h incremental area under the curve, assessed by GC-MS, with two classes for feature selection: samples after cheese intake (class 1) and milk/soy drink intake (class 2). Score plot (a), permutation test (b) and loading plot (c). Discriminating features (VIP > 2) are indicated in red on the loading plot, with indication regarding their identities. Figure S5: OPLS-DA of postprandial serum samples using 6 h incremental area under the curve, assessed by GC-MS, with two classes for feature selection: samples after soy intake (class 1) and milk/cheese intake (class 2). Score plot (a), permutation test (b) and loading plot (c). Discriminating features (VIP > 2) are indicated in red on the loading plot, with indication regarding their identities. Table S1: Selected features discriminating milk, cheese, and soy drink intake based on VIP > 2.0 from OPLS-DA and subsequent univariate analysis based on targeted evaluation from GC-MS data using retention index (RI), quantifier and qualifier ion. Only compounds that have a significant difference (6h-iAUC) between foods are listed. The level of identification (LI) of the metabolites are defined according to Sumner et al. [5]. Table S2: Univariate analysis based on GC-MS targeted evaluation of 6h-iAUCw using retention index (RI), quantifier and qualifier ion from selected candidate markers discriminating milk, cheese, and soy drink intake based on literature or previous knowledge from urine samples [25]. Compound names are shaded in grey if the univariate analysis was significant (*p* < 0.05). Significance was calculated based on the 6h-iAUC of the metabolites except for guaiacol and catechol for which the 24h-iAUC was used. The level of identification (LI) of the metabolites are defined according to Sumner et al. [5]. Table S3: Kruskal-Wallis sum rank test of markers discriminating milk, cheese, and soy drink intake as assessed by GC-MS at each time point. Significant differences between foods (milk intake, cheese intake, soy drink intake) are shaded in grey. Table S4: Multiple comparison test Conover-Inman of markers discriminating milk, cheese, and soy drink intake as assessed by GC-MS at each time point (A = milk intake, B = cheese intake, C = soy drink intake). Significant differences between foods are shaded in grey. Table S5: Fatty acid content [g] from total lipids of test foods per amount served.
