# Supplementary material for: GC-MS Based Metabolomics and NMR Spectroscopy Investigation of Food Intake Biomarkers for Milk and Cheese in Serum of Healthy Humans
Source: Metabolites. 2018 Mar 23;8(2):26. doi: 10.3390/metabo8020026 (PMC6027507; doi:10.3390/metabo8020026)
Supplement: Supplementary file 1 [file metabolites-08-00026-s001.pdf]

Supplementary

GC-MS Based Metabolomics and NMR Spectroscopy Investigation of Food Intake Biomarkers for Milk and Cheese in Serum of Healthy Humans

Alessia Trimigno [1,‡], Linda Münger [2,‡], Gianfranco Picone [1], Carola Freiburghaus [2], Grégory Pimentel [2], Nathalie Vionnet [3], François Pralong [3], Francesco Capozzi [1], René Badertscher [2] and Guy Vergères [2,*]

[1] Department of Agricultural and Food Sciences (DISTAL), University of Bologna, Cesena 47521, Italy; alessia@food.ku.dk (A.T.); gianfranco.picone@unibo.it (G.P.); francesco.capozzi@unibo.it (F.C.)

[2] Agroscope, Berne 3003, Switzerland; muenger.linda@gmail.com (L.M.); carola.freiburghaus@agroscope.admin.ch (C.F.); rene.badertscher@agroscope.admin.ch (R.B.); gregory.pimentel@agroscope.admin.ch

[3] Service of Endocrinology, Diabetes and Metabolism, Lausanne University Hospital, 1011 Lausanne 1005, Switzerland; Nathalie.Vionnet@chuv.ch (N.V.); francois.pralong@latour.ch (F.P.)

‡ The authors contributed equally

* Correspondence: guy.vergeres@agroscope.admin.ch; Tel.: +41-58-463-8154

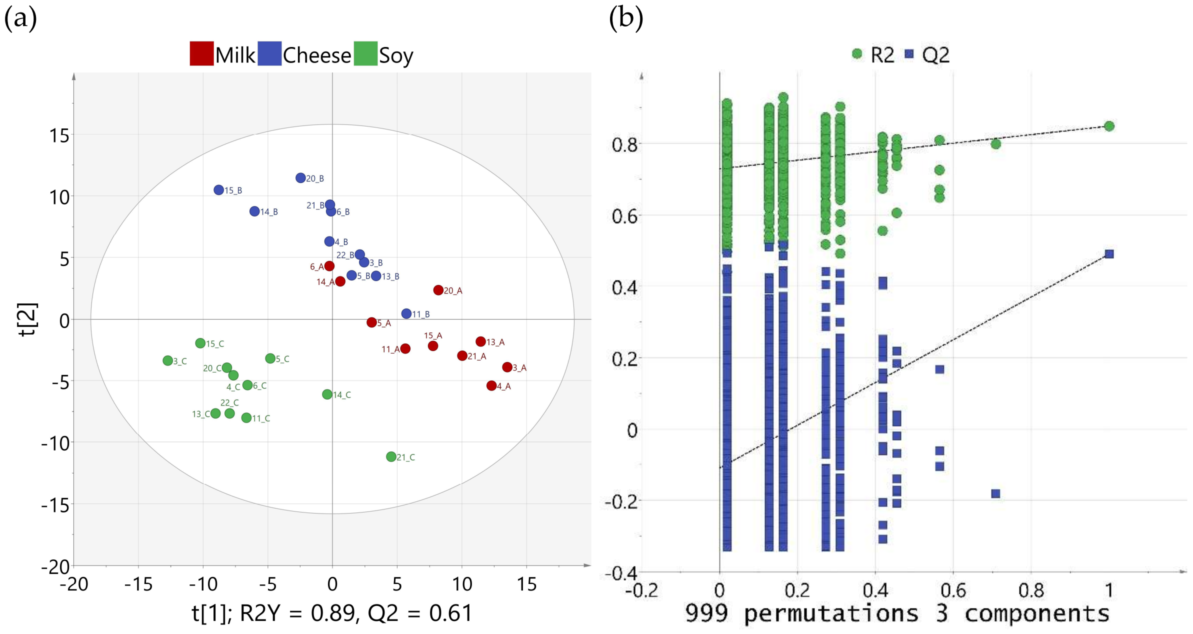

(c)

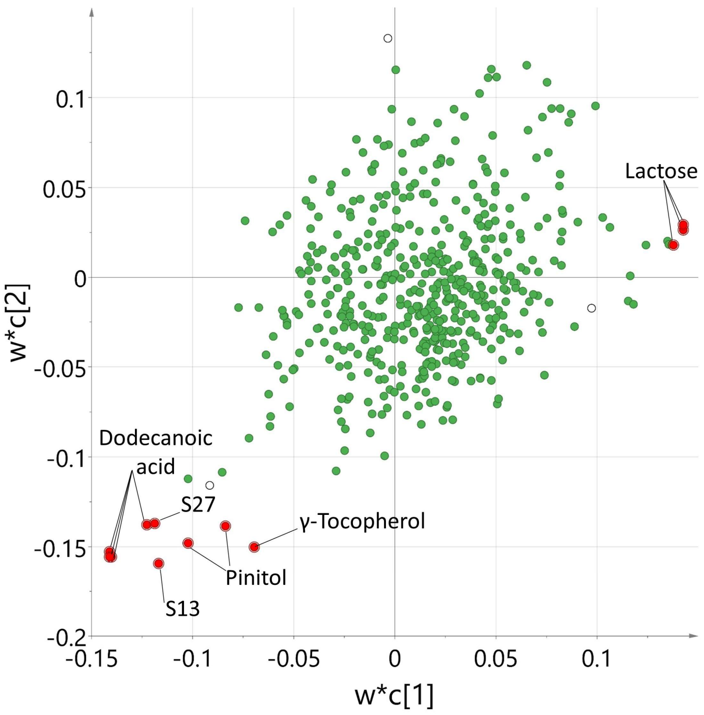

Figure S1. PLS-DA of postprandial serum samples using 6 h incremental area under the curve, with foods as classes (milk intake, cheese intake, and soy drink intake) assessed by GC-MS. Score plot (a), permutation test (b) and loading plot (c). Discriminant features (VIP > 2) are indicated in red on the loading plot, with indication regarding their identities.

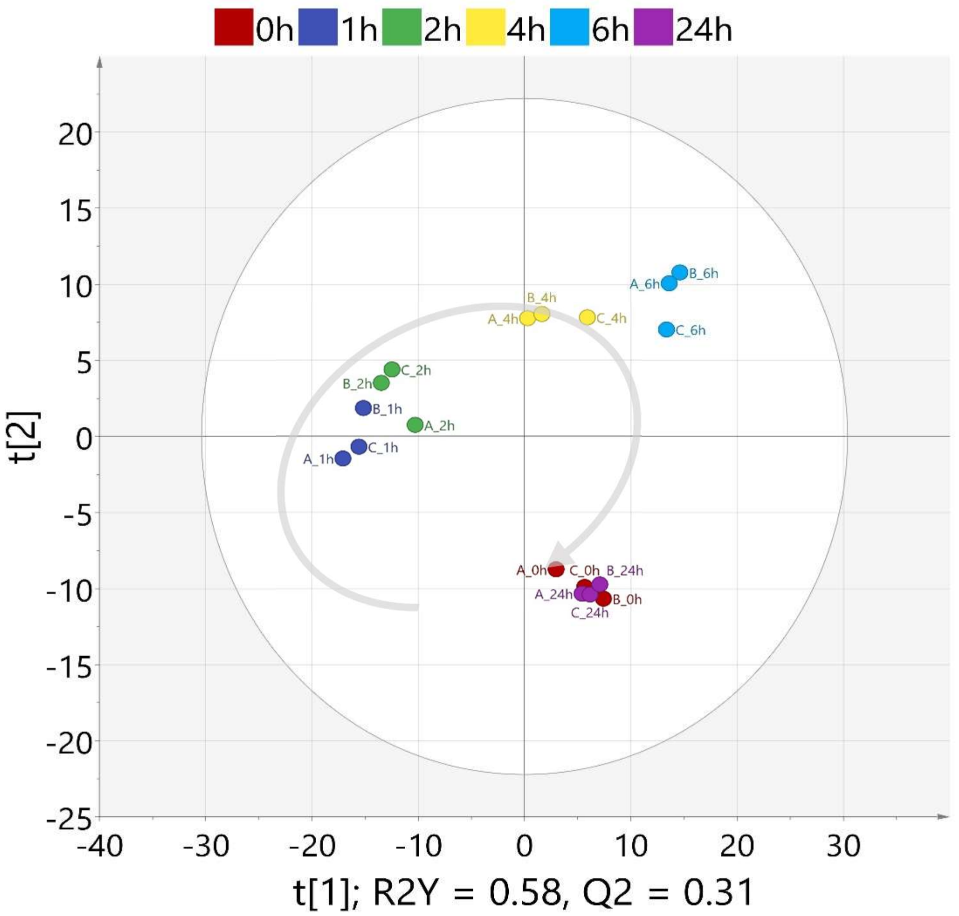

Figure S2. PLS-DA score plot of serum samples after milk intake, cheese intake, and soy drink intake assessed by GC-MS, with time as classes. Median of the 11 subjects was used for each time point and each food.

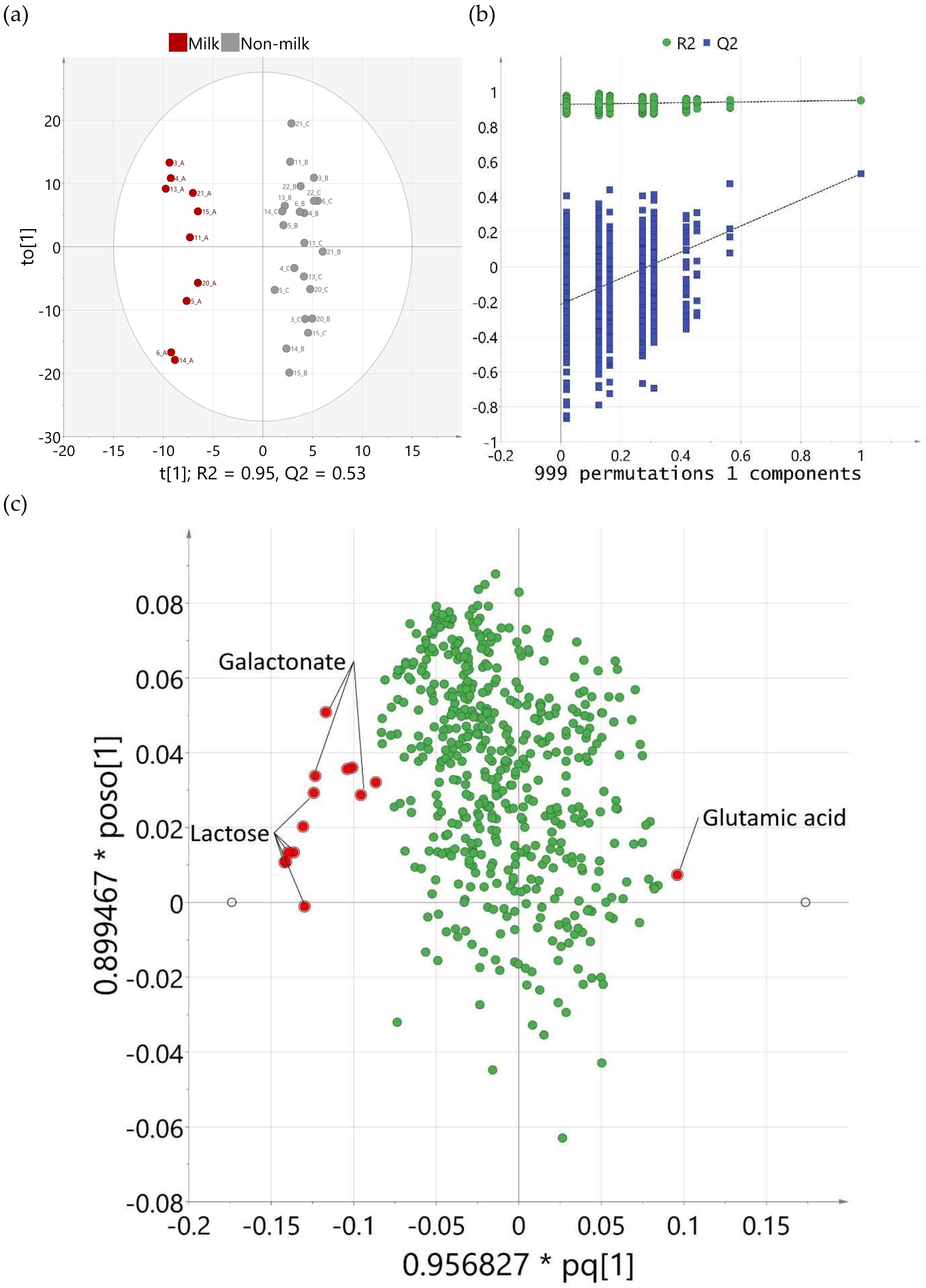

Figure S3. OPLS-DA of postprandial serum samples using 6 h incremental area under the curve, assessed by GC-MS, with two classes for feature selection: samples after milk intake (class 1) and cheese/soy drink intake (class 2). Score plot (a), permutation test (b) and loading plot (c). Discriminating features (VIP > 2) are indicated in red on the loading plot, with indication regarding their identities.

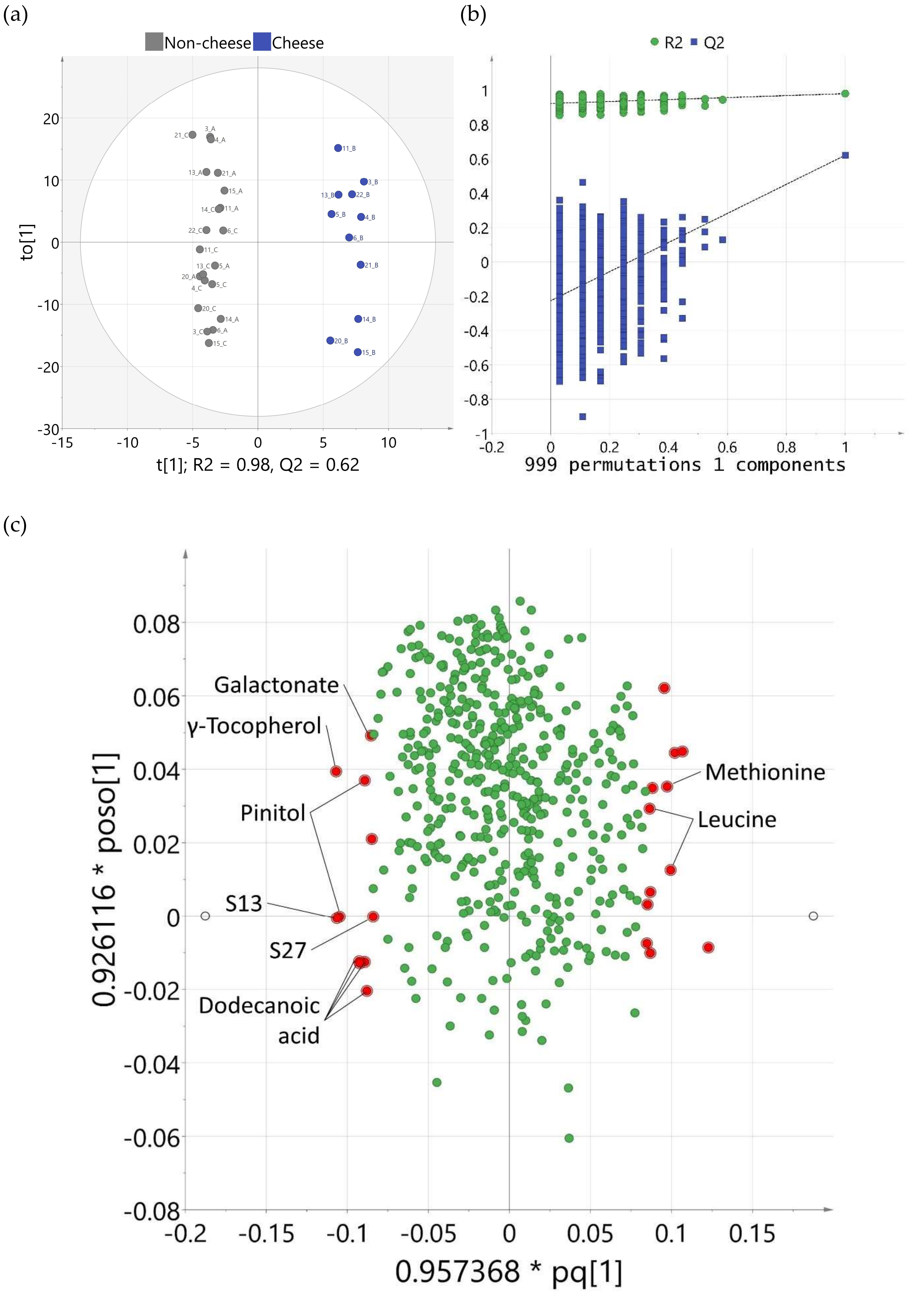

Figure S4. OPLS-DA of postprandial serum samples using 6 h incremental area under the curve, assessed by GC-MS, with two classes for feature selection: samples after cheese intake (class 1) and milk/soy drink intake (class 2). Score plot (a), permutation test (b) and loading plot (c). Discriminating features (VIP > 2) are indicated in red on the loading plot, with indication regarding their identities.

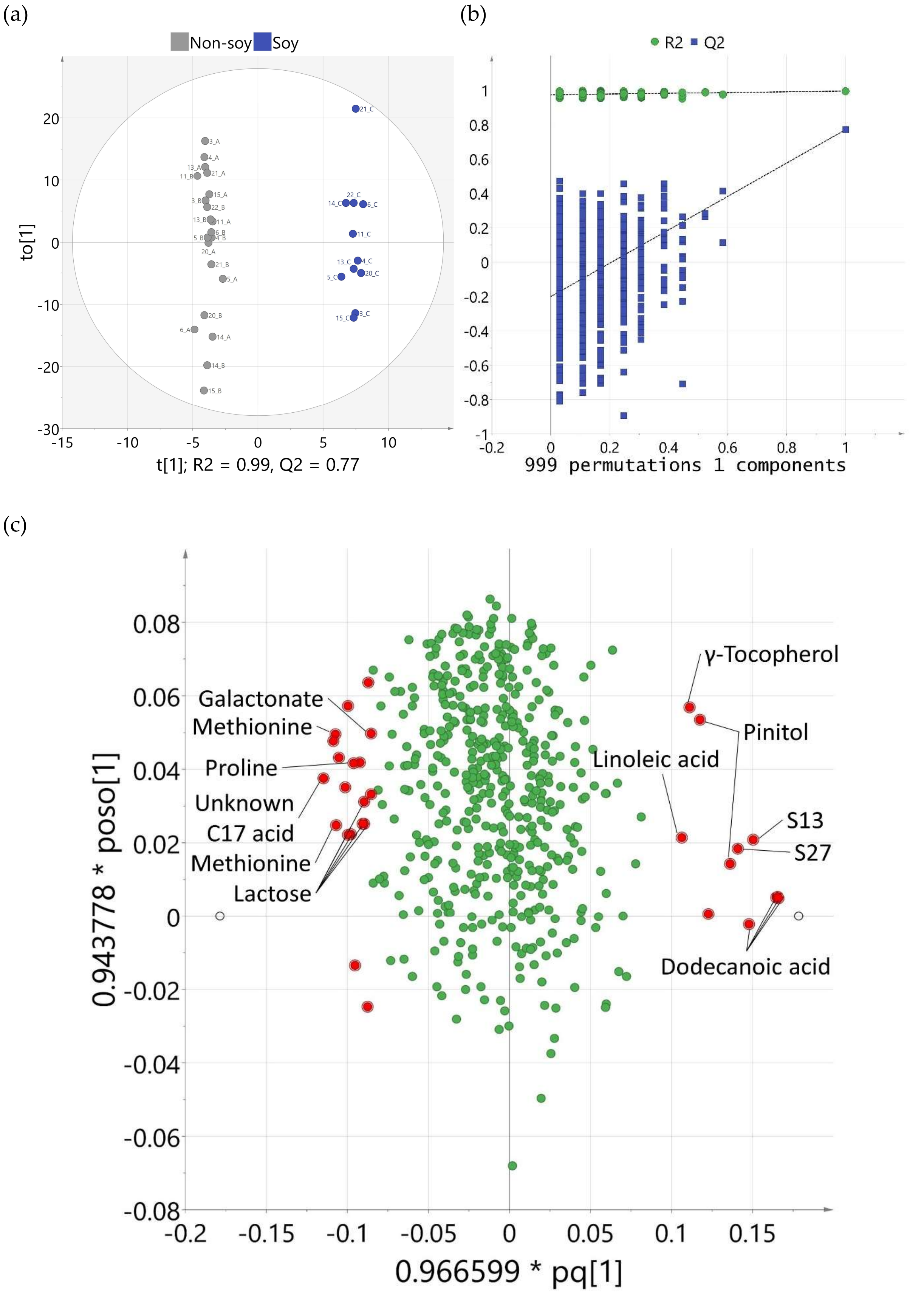

Figure S5. OPLS-DA of postprandial serum samples using 6 h incremental area under the curve, assessed by GC-MS, with two classes for feature selection: samples after soy intake (class 1) and milk/cheese intake (class 2). Score plot (a), permutation test (b) and loading plot (c). Discriminating features (VIP > 2) are indicated in red on the loading plot, with indication regarding their identities.

Table S1. Selected features discriminating milk, cheese, and soy drink intake based on VIP >2.0 from OPLS-DA and subsequent univariate analysis based on targeted evaluation from GC-MS data using retention index (RI), quantifier and qualifier ion. Only compounds that have a significant difference (6h-iAUC) between foods are listed (Kruskal-Wallis, adjusted using the Benjamini Hotchberg correction for multiple testing). The level of identification (LI) of the metabolites are defined according to Sumner et al. [5].

Deconvolution ID	VIP	RI (sample)	RI (reference)	Quantifier ion	Qualifier ion	Ratio	Compound identification	p-value (6h-iAUC)	LI
1640	3.36	2670	2671	361	204	140	Lactose RT 37.30* (M03)	2.74E-04	1
1641	3.33								
1643	3.07								
1644	2.94								
1646	3.23	2690	2691	361	204	140	Lactose RT 37.48* (M04)	2.20E-04	1
1647	3.31								
1636	3.09	*	2641	361	436	40	Lactose RT 37.02*(M26)	1.69E-04	2
1319	2.02	1980	1981	305	319	150	Galactonate	5.90E-05	1
1322	2.27								
1323	2.76								
1326	2.03								
1327	2.03								
800	2.53	1411	1410	104	221	20	Methionine	1.35E-02	1
805	2.54								
660	2.54	1292	1293	142	216	7.2	Proline	1.71E-04	1
611	2.06	1263	1264	158	232	4	Leucine	4.26E-03	1
932	2.27	1527	1527	174	158	20	Glutamic acid	2.80E-02	
1038	3.51	1652	1649	257	132	40	Dodecanoic acid	8.09E-05	1
1047	3.91								
1048	3.92								
1049	3.93								
1454	2.52	2208	2210	337	262	50	Linoleic acid	1.66E-03	1
1461	2.26								
1735	2.63	*		223	488	100	γ-Tocopherol	3.10E-04	2
1203	3.23	1819	1820	260	318	65	Pinitol	5.57E-05	1
1211	2.79								
1692	3.56	*		204	217	32	Unknown (S13)	8.09E-05	4
1125	3.34	1737		217	306	25	Unknown (S27)	8.09E-05	4
1407	2.72	2101		327	132	50	Unknown C17 acid	6.63E-04	3

*no RI calculated as compound was eluting later than alkanes

Table S2. Univariate analysis based on GC-MS targeted evaluation of 6h-iAUCw using retention index (RI), quantifier and qualifier ion from selected candidate markers discriminating milk, cheese, and soy drink intake based on literature or previous knowledge from urine samples [25]. Compound names are shaded in grey if the univariate analysis was significant ($p<0.05$) (Kruskal-Wallis, adjusted using the Benjamini Hotchberg correction for multiple testing). Significance was calculated based on the 6h-iAUC of the metabolites except for guaiacol and catechol for which the 24h-iAUC was used. The level of identification (LI) of the metabolites are defined according to Sumner et al. [5].

RI (sample)	RI (reference)	Quantifier	Qualifier	Ratio	Compound	p-value(6h-iAUC)	LI
1943	1942	299	145	30	Pentadecanoic acid	3.56E-02	1
2139	2138	327	132	50	Heptadecanoic acid	5.77E-02	1
1875	1876	205	319	100	Galactose	7.79E-06	1
1926	1929	217	319	60	Galactitol	3.80E-05	1
1946	1944	319	217	21.7	Galactono-1,5-lactone	8.09E-05	1
1287	1285	183	184	14	Maltol	8.09E-05	1
1581	1580	193	220	43	3-Phenyllactic acid	8.09E-05	1
*	2616	361	217	30	Sucrose	1.34E-02	2
1225	1224	166	181	35	Guaiacol	4.85E-02	1
1313	1314	254	239	26	Catechol	8.83E-02	1

*no RI calculated as compound was eluting later than alkanes

Table S3. Kruskal-Wallis sum rank test of markers discriminating milk, cheese, and soy drink intake as assessed by GC-MS at each time point based on delta values (subtraction of baseline value). Significant differences between foods (milk intake, cheese intake, soy drink intake) are shaded in grey.

	p-value				
	**1h**	**2h**	**4h**	**6h**	**24h**
**Milk**					
Lactose (M03)	8.39E-05	6.71E-05	1.23E-03	1.64E-01	9.52E-01
Lactose (M04)	9.02E-05	6.95E-05	1.23E-03	8.21E-02	9.52E-01
Lactose (M26)	8.39E-05	6.95E-05	8.41E-04	2.02E-01	8.39E-01
Galactose	8.30E-06	8.30E-06	1.56E-01	NA	NA
Galactitol	2.42E-04	1.49E-05	8.65E-05	2.05E-04	5.55E-01
Galactonate	8.39E-05	6.71E-05	8.65E-05	2.05E-04	7.57E-01
Galactono-1,5-lactone	1.24E-01	1.61E-04	2.06E-04	2.23E-04	9.52E-01
**Cheese**					
Methionine	1.89E-03	6.71E-05	1.72E-03	1.23E-01	8.39E-01
Proline	4.71E-02	1.76E-03	1.23E-03	3.82E-02	9.73E-01
Leucine	2.58E-01	8.29E-04	1.07E-02	3.01E-01	9.52E-01
Glutamic acid	2.47E-01	8.92E-03	9.99E-02	1.93E-01	5.88E-01
3-Phenyllactic acid	8.39E-05	6.71E-05	2.27E-03	5.82E-01	8.39E-01
**Soy drink**					
Dodecanoic acid	2.34E-04	6.71E-05	1.27E-04	2.71E-04	8.39E-01
Linoleic acid	5.50E-01	1.44E-02	1.41E-03	7.67E-02	9.60E-01
γ-Tocopherol	3.63E-01	3.72E-02	2.50E-04	6.85E-04	6.70E-01
Pinitol	2.88E-05	1.92E-05	3.04E-05	2.05E-04	7.99E-04
Maltol	8.39E-05	6.71E-05	1.24E-02	4.89E-03	6.70E-01
Sucrose	1.68E-03	2.44E-03	3.86E-01	3.01E-01	6.70E-01
Guaiacol	7.87E-01	4.53E-01	6.17E-01	1.60E-01	5.38E-01
Catechol	7.60E-01	5.84E-01	7.46E-01	8.21E-02	6.70E-01
Unknown (S13)	3.44E-04	6.71E-05	1.27E-04	2.03E-03	9.52E-01
Unknown (S27)	8.33E-02	8.49E-05	1.40E-03	1.97E-03	9.01E-01
**Dairy**					
Pentadecanoic acid	5.53E-01	2.49E-01	1.41EA-02	6.45E-03	9.52E-01
Heptadecanoic acid	8.82E-01	5.29E-01	2.47E-02	2.62E-02	8.29E-01
Unknown C17 acid	4.01E-01	1.07E-01	4.88E-04	1.97E-03	7.57E-01

Table S4. Multiple comparison test Conover-Inman of markers discriminating milk, cheese, and soy drink intake as assessed by GC-MS at each time point (A = milk intake, B= cheese intake, C=soy drink intake) based on delta values (subtraction of baseline value). Significant differences between foods are shaded in grey.

	p-value														
	**1h**			**2h**			**4h**			**6h**			**24h**		
	A vs B	A vs C	B vs C	A vs B	A vs C	B vs C	A vs B	A vs C	B vs C	A vs B	A vs C	B vs C	A vs B	A vs C	B vs C
**Milk**															
Lactose (M03)	6.7E-	6.0E-	2.8E-02	1.6E-	1.8E-	1.3E-01	1.1E-	4.4E-	1.1E-01	2.7E-01	1.1E-	9.8E-	9.8E-01	7.7E-01	9.7E-
Lactose (M04)	1.9E-	1.7E-	3.1E-01	1.9E-	2.8E-	1.6E-01	4.6E-	5.7E-	2.4E-01	1.3E-01	7.2E-	8.9E-	9.8E-01	7.7E-01	8.2E-
Lactose (M26)	3.2E-	8.8E-	9.2E-02	6.8E-	4.3E-	4.6E-01	3.6E-	1.8E-	1.5E-01	2.8E-01	1.5E-	9.8E-	9.8E-01	7.1E-01	8.2E-
Galactose	1.6E-	1.6E-	1.0E+00	1.6E-	1.6E-	1.0E+00	2.1E-	1.2E-	1.0E+00	NA	NA	NA	NA	NA	NA
Galactitol	6.2E-	1.0E-	3.9E-01	1.6E-	4.3E-	5.3E-07	1.3E-	1.7E-	5.4E-03	1.2E-07	1.0E-	2.6E-	3.5E-01	7.1E-01	8.2E-
Galactonate	5.1E-	6.0E-	7.4E-01	3.2E-	1.4E-	1.2E-01	2.6E-	8.2E-	1.9E-01	7.6E-08	3.8E-	1.0E-	9.8E-01	7.1E-01	8.2E-
Galactono-1,5-	1.6E-	5.8E-	8.0E-01	6.2E-	2.4E-	3.4E-01	6.4E-	9.2E-	2.5E-01	9.9E-04	7.7E-	1.5E-	9.8E-01	7.7E-01	9.7E-
**Cheese**															
Methionine	7.1E-	5.3E-	1.6E-04	3.3E-	1.9E-	7.0E-08	5.7E-	1.8E-	2.9E-04	7.2E-01	5.0E-	2.0E-	9.8E-01	7.1E-01	8.2E-
Proline	2.2E-	2.9E-	1.6E-02	1.3E-	7.0E-	4.8E-04	3.6E-	1.8E-	8.4E-05	2.6E-01	3.4E-	1.0E-	9.8E-01	9.4E-01	9.7E-
Leucine	2.4E-	8.6E-	2.1E-01	3.3E-	1.2E-	4.8E-02	3.4E-	5.3E-	2.6E-03	6.2E-01	6.8E-	1.7E-	9.8E-01	7.7E-01	9.7E-
Glutamic acid	1.6E-	2.9E-	7.6E-01	3.0E-	1.4E-	5.6E-01	1.6E-	9.2E-	6.7E-02	2.6E-01	7.9E-	1.8E-	3.5E-01	7.1E-01	8.2E-
3-Phenyllactic acid	6.2E-	2.6E-	2.1E-07	1.8E-	8.5E-	7.8E-07	6.0E-	2.0E-	7.8E-03	7.2E-01	8.4E-	3.6E-	9.8E-01	7.1E-01	9.4E-
**Soy Drink**															
Dodecanoic acid	2.3E-	1.7E-	1.6E-04	9.7E-	6.4E-	8.3E-07	5.7E-	4.4E-	5.7E-06	8.9E-01	3.8E-	2.7E-	9.8E-01	7.8E-01	8.2E-
Linoleic acid	3.7E-	4.7E-	9.4E-01	6.3E-	5.0E-	3.5E-02	6.6E-	1.6E-	1.2E-03	9.9E-01	4.2E-	6.3E-	9.8E-01	7.7E-01	9.7E-
γ-Tocopherol	2.2E-	6.3E-	5.5E-01	9.6E-	4.8E-	1.9E-02	9.5E-	8.8E-	8.7E-06	6.2E-01	3.2E-	2.7E-	8.3E-01	7.7E-01	6.3E-
Pinitol	1.0E-	3.8E-	1.5E-08	6.6E-	6.9E-	3.3E-10	3.7E-	4.1E-	7.4E-11	7.2E-01	2.3E-	5.1E-	9.8E-01	7.4E-06	8.4E-
Maltol	3.8E-	1.4E-	6.4E-06	3.4E-	1.8E-	2.7E-06	9.1E-	8.3E-	7.4E-03	1.0E+00	1.4E-	2.6E-	9.8E-01	7.1E-01	6.3E-
Sucrose	6.7E-	8.6E-	3.2E-04	6.3E-	4.0E-	3.8E-03	6.6E-	1.9E-	4.4E-01	6.2E-01	6.8E-	1.7E-	6.1E-01	7.1E-01	8.2E-
Guaiacol	5.9E-	9.5E-	7.5E-01	6.5E-	2.6E-	5.9E-01	5.7E-	4.2E-	1.0E+00	9.9E-01	1.4E-	9.9E-	9.8E-01	1.9E-01	6.3E-
Catechol	6.7E-	5.7E-	8.4E-01	6.8E-	3.6E-	6.8E-01	6.6E-	9.7E-	5.8E-01	8.9E-01	1.0E-	3.9E-	9.8E-01	7.1E-01	7.1E-
Unknown (S13)	8.7E-	3.5E-	4.5E-05	7.5E-	2.8E-	7.8E-07	5.7E-	4.4E-	5.7E-06	1.0E+00	5.4E-	6.0E-	9.8E-01	1.0E+00	9.4E-
Unknown (S27)	2.4E-	2.7E-	4.9E-01	2.7E-	4.4E-	2.3E-05	4.2E-	8.0E-	3.1E-03	1.0E+00	4.9E-	5.8E-	9.8E-01	7.6E-01	9.7E-
**Dairy**															
Pentadecanoic acid	3.7E-	9.2E-	5.5E-01	7.4E-	2.6E-	1.6E-01	6.6E-	2.0E-	5.7E-03	9.9E-01	1.7E-	4.8E-	9.8E-01	7.7E-01	9.7E-
Heptadecanoic acid	6.8E-	9.5E-	8.0E-01	7.3E-	3.1E-	5.6E-01	8.8E-	2.0E-	1.5E-02	9.9E-01	2.1E-	1.0E-	8.3E-01	7.1E-01	9.7E-
Unknown C17 acid	2.3E-	6.5E-	5.6E-01	4.9E-	2.6E-	5.5E-02	2.1E-	5.4E-	8.7E-06	5.0E-01	2.3E-	1.6E-	9.8E-01	7.1E-01	7.5E-

*ns = not significant

Table S5. Fatty acid content [g] from total lipids of test foods per amount served.

	Milk (600 ml)	Cheese (100 g)	Soy drink (600 ml)
Butyric acid C4:0	0.75	1.02	0.003
Valeric acid C5:0	0.01	0.01	< LOD
Caproic acid C6:0	0.47	0.63	0.04
Enanthic acid C7:0	0.01	0.01	< LOD
Caprylic acid C8:0	0.28	0.37	0.56
Capric acid C10:0 (decanoic acid)	0.64	0.81	0.51
Caproleic acid C10:1	0.08	0.10	< LOD
Lauric acid C12:0 (dodecanoic acid)	0.73	0.89	6.66
Myristic acid C14:0	2.59	3.19	2.27
C14:1 t	0.00	0.00	< LOD
C14:1 c	0.23	0.27	< LOD
Pentadecanoic acid C15:0	0.29	0.34	0.01
Palmitic acid C16:0	7.07	8.78	3.79
trans-Palmitoleic acid C16:1 t	0.03	0.03	< LOD
Palmitoleic acid C16:1 c	0.34	0.42	0.01
Heptadecanoic acid C17:0	0.14	0.19	0.02
trans-Heptadecaenoic acid C17:1 t	0.00	< LOD	< LOD
Stearic acid C18:0	2.13	3.26	3.62
C18:1 t4	0.00	0.01	< LOD
C18:1 t5	0.00	0.01	< LOD
C18:1 t6-8	0.04	0.06	0.005
Elaidic acid (C18:1 t9)	0.06	0.10	0.01
C18:1 t10-11	0.52	0.68	0.01
C18:1 t12	0.06	0.08	0.004
C18:1 t13-14+c6-8	0.15	0.20	< LOD
Oleic acid (C18:1 c9)	3.84	5.91	3.21
C18:1 c11	0.09	0.15	0.16
C18:1 c12	0.04	0.05	0.01
C18:1 c13	0.01	0.02	0.01
C18:1 t16+c14	0.08	0.10	< LOD
C18:2 t9t12	0.00	0.01	< LOD
C18:2 c9t13+(t8c12)	0.05	0.06	0.005
C18:2 c9t12+(c,c-MID+t8c13)	0.06	0.08	0.01
C18:2 t11c15+t9c12	0.08	0.07	0.005
Linoleic acid (C18:2 c9c12)	0.35	0.52	6.35
C18:2 c9c15	0.01	0.01	0.01
C18:3 c9c12c15	0.22	0.23	1.00
Conjugated linoleic acids (C18:2 c9t11+t8c10+t7c9)	0.22	0.26	0.00
Conjugated linoleic acids (C18:2 t11c13+c9c11)	0.02	0.01	< LOD
Conjugated linoleic acid (C18:2 t9t11)	0.01	0.01	0.004
C19:0	0.02	0.02	< LOD
C20:0	0.03	0.06	0.08
C20:1 t	0.01	0.01	< LOD
C20:1 c5	0.00	0.01	< LOD
C20:1 c9	0.03	0.05	< LOD

C20:1 c11	0.01	0.02	0.02
C20:2 c,c (n-6)	0.01	0.01	0.01
C22:0	0.03	0.03	0.05
C20:3 (n-6)	0.01	0.02	< LOD
C20:3 (n-3)	0.00	0.01	< LOD
Arachidonic acid (C20:4, n-6)	0.02	0.03	0.004
Eicosapentaenoic acid (EPA, C20:5)	0.02	0.02	< LOD
C22:5 (DPA) (n-3)	0.03	0.03	< LOD
Docosahexaenoic acid (DHA, C22:6 n-3)	0.00	0.01	< LOD
